# Supplementary material for: The cyanobacterial ESCRT-III protein IM30 forms biomolecular condensates at physiologically relevant conditions
Source: Biophys J. 2026 Jan 12;125(4):1081–94. doi: 10.1016/j.bpj.2026.01.011 (PMC13351666; doi:10.1016/j.bpj.2026.01.011)
Supplement: Document S1. Figures S1–S4 [file mmc1.pdf]

Biophysical Journal, Volume *125*

Supplemental information

The cyanobacterial ESCRT-III protein IM30 forms biomolecular condensates at physiologically relevant conditions

Ndjali Quarta, Tika Ram Bhandari, Katrin Debrich, Nadja Hellmann, Martin Girard, and Dirk Schneider

Supporting information

The cyanobacterial ESCRT-III protein IM30 forms biomolecular condensates at physiologically relevant conditions

Ndjali Quarta,[1] Tika Ram Bhandari,[2] Katrin Debrich[1], Nadja Hellmann,[1] Martin Girard,[2] and Dirk Schneider,[1,3,]*

[1]Department of Chemistry – Biochemistry, Johannes Gutenberg University, 55128 Mainz, Germany

[2]Max Planck Institute for Polymer Research, Ackermannweg 10, Mainz 55128, Germany

[3]Institute of Molecular Physiology, Johannes Gutenberg University, 55099 Mainz, Germany

* Correspondence: Dirk.Schneider@uni-mainz.de

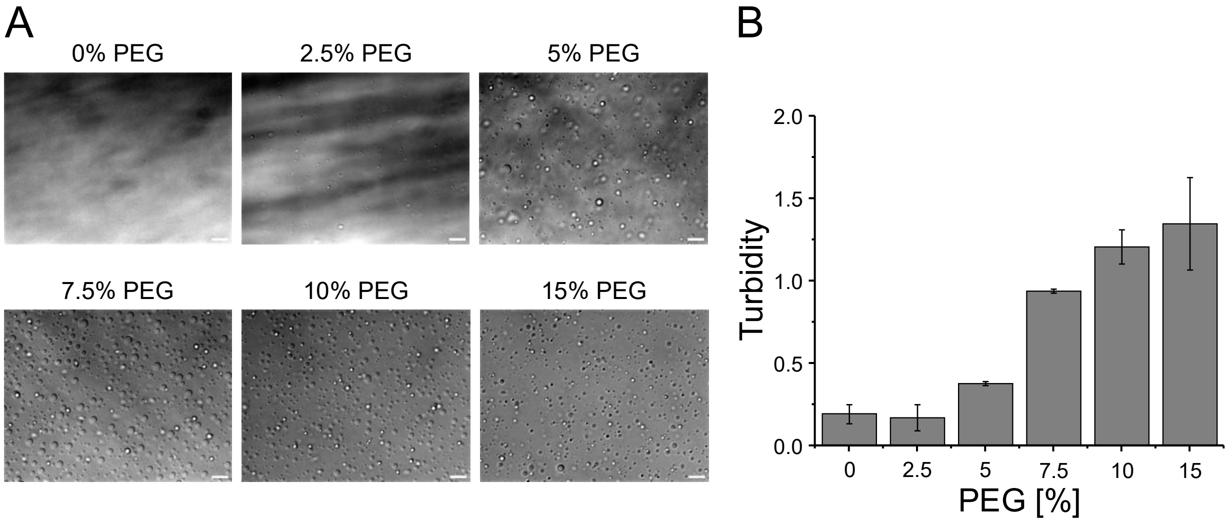

Figure S1: Phase separation and the formation of IM30* condensates at increasing PEG concentrations.

Increasing PEG amounts were added to 32 µM IM30* dissolved in 20 mM HEPES buffer (pH 7.6) containing 100 mM NaCl. The samples were incubated for 5 min. before (A) condensate formation was visualized via DIC microscopy and (B) the turbidity of the solution was measured. At 5% PEG the formation of condensates was already observed, which however, was more pronounced at higher PEG concentrations. Scale bar in (A): 10 µm

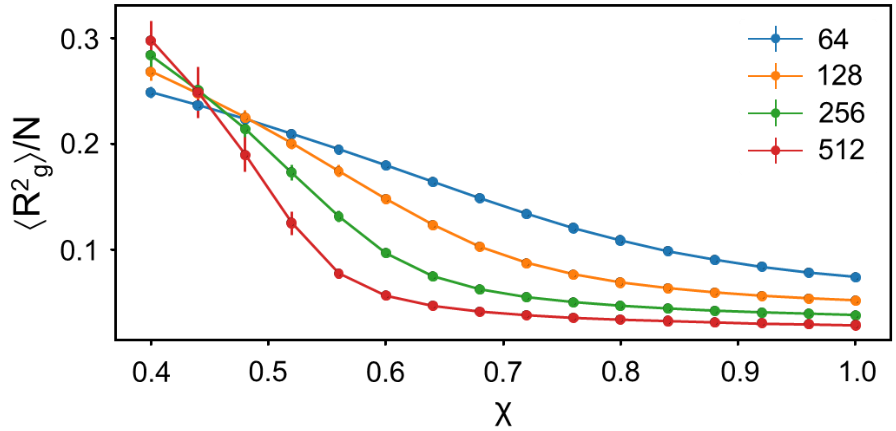

Figure S2: Estimation of the polymer collapse temperature.

The theta point is a special temperature at which a polymer chain in solution behaves ideally, meaning that the chain neither collapses nor expands due to interactions with the surrounding solvent. It also corresponds to the temperature at which an infinite chain goes from an extended conformation to a collapsed one, and, therefore, corresponds to the polymer collapse temperature. At this point, the attractive and repulsive forces between different parts of the chain balance out perfectly.

The theta point can be estimated via computer simulations of chains of different lengths and measuring their radius of gyration, which reflects how spread out the chain is, at different temperatures. When this size measure is normalized and plotted for various chain lengths, the temperature at which the curves cross indicates the theta point. Curves for simulations using different values of a parameter called the Ashbaugh-Hatch scale are shown. The beads are modeled with a size of 6 Å. For these settings, the theta temperature is found to be around 0.45. It’s important to note that this value isn't universal, it rather depends on the chemical details of the polymer, especially the size and nature of its side chains. Changing these features can shift the theta point, so the result is specific to the system being modeled.

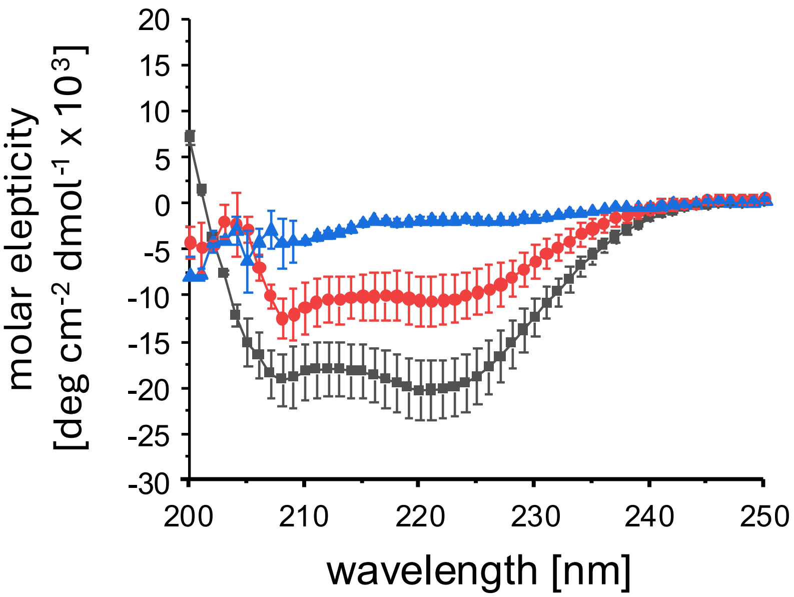

Figure S3: The structure of IM30 wt at increasing urea concentrations.

IM30 wt was incubated in pure buffer (10 mM HEPES pH 7.6; black) or in buffer containing 3 m (red) or 6 M (blue) urea, respectively. After 30 min. incubation, CD spectra were measured. The mean ±SD of three independent measurements (independent protein purifications) is shown. While some α-helix structure is retained at 3 M urea, which corresponds to the helical-hairpin (44), at 6 M urea the protein is completely unfolded.

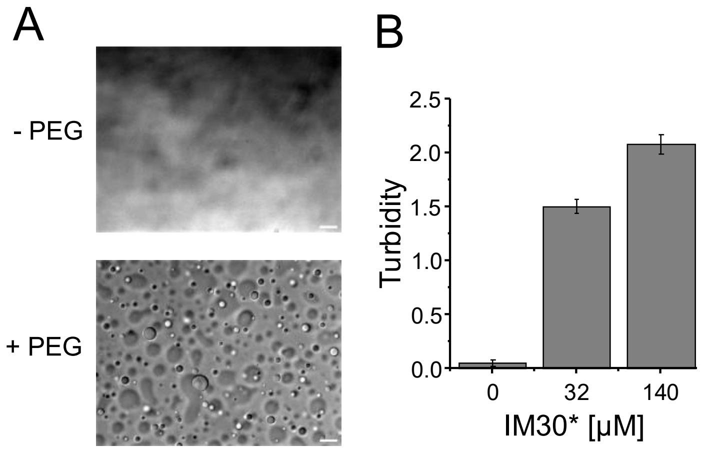

Figure S4: Condensate formation of 140 µM IM30*.

The maximum intracellular IM30 concentration was calculated to be ~140 µM (see main text for details). Even at such high protein concentrations the protein phase separates and form condensates when PEG is present. (A) DIC microscopy images of 140 µM IM30* in 20 mM HEPES buffer (pH 7.6), 100 mM NaCl +/- 10 % PEG. Scale bar: 10 µM. (B) Turbidity of IM30 solutions in the presence of 100 mM NaCl and 10% PEG. Turbidity values for 0, 32 (compare Figure 6 in the main text) and 140 µM protein are shown (n=3 ±SD).
