# Supplementary material for: The cyanobacterial ESCRT-III protein IM30 forms biomolecular condensates at physiologically relevant conditions
Source: Biophys J. 2026 Jan 12;125(4):1081–94. doi: 10.1016/j.bpj.2026.01.011 (PMC13351666; doi:10.1016/j.bpj.2026.01.011)
Supplement: Document S2. Article plus supporting material [file mmc2.pdf]

Biophysical Journal
Article

Biophysical Society

The cyanobacterial ESCRT-III protein IM30 forms biomolecular condensates at physiologically relevant conditions

Ndjali Quarta,[1] Tika Ram Bhandari,[2] Katrin Debrich,[1] Nadja Hellmann,[1] Martin Girard,[2] and Dirk Schneider[1,3,*]
[1]Department of Chemistry – Biochemistry, Johannes Gutenberg University, Mainz, Germany; [2]Max Planck Institute for Polymer Research, Mainz, Germany; and [3]Institute of Molecular Physiology, Johannes Gutenberg University, Mainz, Germany

ABSTRACT IM30, the inner membrane-associated protein of 30 kDa, conserved in cyanobacteria and chloroplasts, is a member of the ESCRT-III superfamily of membrane remodeling proteins. Like other ESCRT-III proteins, IM30 forms higher-order oligomeric structures, although the mechanisms regulating its assembly and disassembly remain poorly understood. A hallmark of ESCRT-III protein monomers is the presence of at least five α-helices, with the long helices α1 and α2/3 forming a helical hairpin that constitutes the structural core of all superfamily members. In contrast to eukaryotic ESCRT-III subunits, helices α0 and α4-α6 of *Synechocystis* IM30 unfold upon oligomer disassembly. Given that intrinsically disordered proteins often form biomolecular condensates via liquid-liquid phase separation and IM30 has previously been observed to form puncta structures in vivo under membrane stress, we here investigated whether IM30 has the ability to form biomolecular condensates in vitro. We demonstrate that IM30 forms condensates under physiologically relevant conditions of salt and protein concentrations, suggesting a functional link between the now observed condensate formation and membrane dynamics. Condensate formation is driven by the polyampholyte nature of IM30, yielding condensates that can be dissolved by both high and low salt concentrations. In living cyanobacterial cells, we observed puncta structures under salt stress, which we now link to the formation of condensates. We propose that condensates serve as transient hubs, locally concentrating IM30 monomers under stress conditions without requiring energy-intensive disassembly of preformed oligomers. Thus, condensate formation may represent a crucial early step in IM30-mediated stress response in bacteria and chloroplasts.

SIGNIFICANCE IM30, a protein conserved in cyanobacteria and chloroplasts, mediates membrane remodeling. As a homologous eukaryotic (ESCRT-III) protein, it forms oligomers. Some α-helices of IM30 become disordered upon oligomer disassembly, and we now show that monomeric IM30 forms liquid-like, highly dynamic, membraneless structures (condensates) in vitro, which form via separation of the proteins into a high-density phase. The formation of such structures is sensitive to ionic strength. Given that IM30 forms puncta structures in vivo at salt stress conditions, we propose that condensates act as transient hubs, concentrating IM30 monomers, which facilitates formation of oligomeric structures at specific intracellular locations. Thus, stress-induced condensate formation likely represents a regulatory step in IM30-mediated stress response in prokaryotes and chloroplasts.

INTRODUCTION

IM30, the inner membrane-associated protein of 30 kDa, also known as Vipp1 (vesicle-inducing protein in plastids 1), is conserved in chloroplasts and cyanobacteria (1–3). In contrast to most bacteria, cyanobacteria possess not only a cytoplasmic membrane but also an additional internal membrane system, the thylakoid membranes (TMs), where photosynthetic electron transfer reactions take place (4,5). This is also a feature of chloroplasts, which contain TMs in addition to the inner envelope membrane. The shared presence of TMs reflects the evolutionary ancestry between cyanobacteria and chloroplasts, supporting their common origin through endosymbiosis (6,7).

The protein IM30 likely evolved via gene duplication from a gene coding for the phage shock protein A (PspA), found in many bacterial lineages, including cyanobacteria (1–3,8,9). In these bacteria, PspA appears to be involved in maintaining, stabilizing, and/or repairing the cytoplasmic

Submitted September 25, 2025, and accepted for publication January 8, 2026.

*Correspondence: dirk.schneider@uni-mainz.de

Editor: Samrat Mukhopadhyay.
https://doi.org/10.1016/j.bpj.2026.01.011

© 2026 The Author(s). Published by Elsevier Inc. on behalf of Biophysical Society.
This is an open access article under the CC BY license (http://creativecommons.org/licenses/by/4.0/).

Check for updates

membrane. Similarly, IM30 appears to play a crucial role in the biogenesis, dynamics, maintenance, stabilization, and/or repair of internal membranes in chloroplasts and cyanobacteria (3,10–18).

In 2021, the structures of two cyanobacterial IM30s and a cyanobacterial PspA were solved, revealing their relation to the endosomal sorting complex required for transport-III (ESCRT-III) protein superfamily, previously thought to be exclusively present in eukaryotes and archaea (19–21). The monomer structures of all ESCRT-III superfamily members are conserved, with five α-helical regions connected by short linkers (10,22). The extended helices α1 and α2 form a helical hairpin, and the following short helix α3 is a direct extension of α2 when the protein is in an open conformation. Helices α4 and α5 are critical for intermonomer contacts in oligomeric assemblies. Several ESCRT-III superfamily members, including PspA and IM30, have an additional N-terminal helix α0 crucially for membrane interaction (12,20,21,23,24). While bacterial PspA contains only six helices (α0-α5), cyanobacterial and chloroplast IM30 have an extra C-terminal helical region, helix α6, separated from α5 by an extended, unstructured linker. The function of this helix is largely enigmatic, yet it appears to be crucial for IM30's in vivo activity (25–27). Eukaryotic ESCRT-III proteins often have additional C-terminal domains with defined functions, such as protein-protein interactions (22,28–30).

All ESCRT-III superfamily members exhibit a high propensity for forming higher-ordered oligomeric structures. PspA and IM30 form large homo-oligomeric supercomplexes, whereas eukaryotic ESCRT-III typically hetero-oligomerizes (22,28). Cyanobacterial IM30 exists as free monomers (or small oligomers) in solution, which are capable of forming barrels and rods in solution plus polymeric membrane-attached structures, such as carpets, spirals, or membrane-bound barrels/rods, as mainly demonstrated by in vitro analyses (12,13,20–22,31–37). The membrane-attached IM30 barrel or rod structures are capable of internalizing membranes, resulting in tubulated membrane structures (12,20,21). Yet, the mechanisms governing IM30 oligomer assembly remain poorly understood. In contrast to the prokaryotic system, eukaryotic ESCRT-III oligomer assembly at a membrane is orchestrated by other ESCRT family members (ESCRT-0 to ESCRT-II) (28,30,38–40). In solution, monomeric eukaryotic ESCRT-IIIs adopt a closed conformation, where α3 and α4 pack against the open end of the α1/α2 helical hairpin, and α5 folds back, auto-inhibiting membrane binding and oligomerization (41–43). The open conformation, representing the polymerization-competent state, involves α3 extending from α2 (as also observed in PspA and IM30), and α4 and α5 detaching for intramonomer interactions. Unlike eukaryotic ESCRT-III subunits, a closed conformation has not been observed for bacterial PspA and IM30 proteins yet. In contrast, helices α0 and α4-6 of the IM30 protein of the cyanobacterium *Synechocystis* sp. PCC 6803 (from here on: *Synechocystis*) appear to unfold upon monomerization (33,44).

Although prokaryotic and eukaryotic ESCRT-III oligomer assemblies differ in shape, their structural organization is remarkably similar. In oligomeric assemblies, each ESCRT-III monomer interacts with multiple neighboring subunits, with α5 of one monomer packing perpendicularly against the α1-3 hairpin of a neighboring monomer. Additional contacts, such as between α4 of one monomer and the α1-3 hairpin of a neighboring protomer and parallel stacking of α1-3 hairpins, further organize and stabilize these assemblies (22), finally resulting in the formation of barrel-like structures and higher-order rod-shaped complexes with masses of several MDa (22,30).

When IM30 of *Arabidopsis thaliana* was highly overexpressed in tobacco plants, storage of overexpressed IM30 as bundled filaments was observed (45). However, at near-native expression levels, bacterial ESCRT-III proteins (e.g., PspA of *E. coli*, LiaH of *B. subtilis*, and IM30 of *Synechocystis*) are evenly distributed within the bacterial cytoplasm, yet form puncta structures near internal membranes under membrane stress conditions (46–51). Similar structures have been observed for the chloroplast homolog, although their nature remains elusive (18,26). All ESCRT-III superfamily members appear to be implicated in membrane stabilization and/or repair (10,13,15,24,28,29,38,39,52). Given that a significant fraction of *Synechocystis* IM30 is disordered when the protein is monomeric (33,44) and that intrinsically disordered proteins (IDPs) or proteins with intrinsically disordered regions (IDRs) often form biomolecular condensates via phase separation (53–55), we now investigated whether the cyanobacterial protein has the potential to form condensates under physiologically relevant conditions.

Biomolecular protein condensates are dynamic, liquid-like droplets forming when a protein solution undergoes phase separation, resulting in regions of high protein concentration (the condensates) coexisting with regions of low protein concentration. Protein condensates can be described as viscoelastic, networked fluids that form via phase separation of macromolecules coupled to percolation (56,57). For most IDPs, the process of protein phase separation can be understood through the perspective of polymer physics. If hydrophobic contributions are dominant, the protein behavior can be understood as the result of a single quantity: the solvent quality χ, a measure of how favorably polymer chains interact with the surrounding solvent. Solvent quality is not an intrinsic property but is highly sensitive to environmental conditions, such as salt concentration, pH, temperature, and molecular crowding. For polymers, including polypeptide chains, the minimal concentration needed for phase separation, the critical saturation concentration c_{sat}, scales with the degree of polymerization N (i.e., the number of monomeric units in the chain) as $N^{-1/2}$, thus longer chains exhibit lower c_{sat} values. This model describes well mostly neutral, low-complexity chains, as, e.g., found in

the well-studied protein FUS (58,59). Yet, in contrast to a dominating hydrophobic contribution, the electrostatic behavior of polyampholytes, i.e., polymers containing both positively and negatively charged monomers, is much more complex, particularly when the charge asymmetry is low. In such quasineutral polyampholytes, long-range electrostatic attractions between oppositely charged segments can dominate over repulsive forces, leading to intramolecular or intermolecular association. However, unlike a purely hydrophobic collapse, which typically results in compact globules, the spatial arrangement and interactions of charged groups in polyampholytes can give rise to nonuniform, heterogeneous morphologies, such as pearl-necklace architectures (see (60,61) and references therein). Moreover, the phase behavior of polyampholyte solutions exhibits a hallmark feature known as re-entrant phase separation with respect to salt concentration. That is, phase separation does not occur at very low or very high salt levels, but only at intermediate salt concentrations, linked to competition between polyelectrolyte and polyampholyte behaviors (62).

We now observed that IM30 phase separates in vitro under physiologically relevant salt and protein concentrations, indicating a potential functional role of this process in cells. Condensate formation is driven by IM30's polyampholyte character and is disrupted at both high and low ionic strengths. We suggest that the condensates act in vivo as transient hubs, concentrating IM30 monomers at defined intracellular sites without the need for energy-dependent disassembly of preformed oligomers. In fact, in living cyanobacterial cells IM30 forms puncta structures when cells are salt-stressed, which links the condensates that form in vitro with the protein's in vivo behavior. Thus, phase separation potentially represents an early, key step in IM30-mediated stress response, linking protein condensation to the dynamics of cyanobacterial and chloroplast inner membrane systems.

MATERIAL AND METHODS

Cloning, expression, and purification of IM30 variants for in vitro studies

Construction of the plasmids used to express the genes coding for *Synechocystis* sp. PCC 6803 *im30* and *im30** has been described previously (33,34). In contrast to the wild-type (WT) protein, IM30* contains six mutations: E83A, E84A, F168A, E169A, R170A, and M171A. For IM30_mVenus, a Gly-Ser linker of seven amino acids was added after the IM30 WT C-terminus before the mVenus fluorescence tag, which has previously been shown to result in a functional fusion protein (47). The sequences of all the plasmids were confirmed by sequencing (Eurofins, Ebersberg, Germany).

For the in vitro analyses, the protein variants were expressed in *E. coli* BL21 (DE3) grown overnight in LB medium at 37°C. Cells were harvested by centrifugation (3000 × *g*, 4°C), resuspended in buffer (300 mM NaCl, 20 mM imidazole, 50 mM phosphate [pH 7.6]) and lysed by sonication at 4°C. Cell debris was removed by centrifugation (12,000 × *g*, 4°C), and proteins bound to a Ni-NTA column were washed with increasing amounts of imidazole (20, 50, or 100 mM). His-tagged proteins were finally eluted with a buffer containing 500 mM imidazole. The buffer was exchanged to 10 mM phosphate, 10 mM HEPES (pH 7.6) using PD-10 columns or dialysis, and the proteins were concentrated using centrifugal filters (Merck, Darmstadt, Germany) with a molecular weight cut-off of 30 kDa for IM30 and IM30-mVenus, and 10 kDa for IM30* and IM30*-mVenus, respectively. Protein concentrations were determined with a Bradford assay using bovine serum albumin (BSA) as a standard for the calibration curve, and the proteins were frozen in liquid nitrogen and stored at −20°C until use. To validate the purity of aliquoted protein samples, proteins (1 μg/lane) were separated on a 12% SDS-PAGE gel and stained after electrophoresis with Coomassie brilliant blue R250.

Circular dichroism spectroscopy

Urea-induced alterations in the secondary structure of IM30 were monitored via circular dichroism (CD) spectroscopy. For each data point, a protein solution at a concentration of 0.1 mg mL^{-1} was preincubated for 30 min at 20°C in 10 mM HEPES buffer (pH 7.6) containing the indicated urea concentration. CD spectra were then recorded at 20°C using a JASCO J-1500 spectropolarimeter (JASCO, Tokyo, Japan), scanning the wavelength range from 190 to 250 nm with a step size of 1 nm, a scan rate of 100 nm min^{-1}, and a slit width set to 1 nm. Each condition was measured at least three times independently, and the reported spectra correspond to the average of these replicates.

Turbidity measurements and phase diagrams

Turbidity measurements were performed in 384-well plates (Cellvis, Mountain View, CA) using an Omega plate reader (BMG LABTECH, Ortenberg, Germany). Protein samples (60 μL) with the specified concentrations were prepared in either phosphate buffer (10 mM phosphate, 10 mM HEPES [pH 7.6]) or PEG/NaCl buffer (20 mM HEPES [pH 7.6], 10% [w/v] PEG-8000, NaCl as indicated) at the indicated urea concentrations. To ensure well-mixed and rapidly equilibrated protein solutions, the proteins were premixed with 4 M urea if applicable. The premixed protein was then added to the respective buffer to reach the final composition for the experiment. Fifty microliters were transferred to a 384-well plate and incubated for 5 min at room temperature, in which phase separation was completed. Subsequently, the turbidity was measured as the absorbance value at 350 nm at room temperature. Data from three independent turbidity measurements were combined to visualize condensate-forming conditions in the phase diagram. Here, onset of phase separation was defined to occur at an absorption of 0.13, and strong phase separation above twice that value, namely 0.26.

Differential interference contrast and fluorescence microscopy of condensates

Samples were prepared as described above for the turbidity measurements and imaged after 15 min incubation time using an Axio Observer.Z1 (Carl Zeiss, Jena, Germany) equipped with a 63× oil objective in differential interference contrast (DIC) mode, 500 ms acquisition time, and 4.5–5.5 V lamp voltage. In the case of fluorescently labeled crowders, 12.5 μM (1 mol% in case of 10% PEG-8000) Cy5-PEG (Biopharma PEG Scientific, Watertown, MA) was added to the PEG mixture for condensate formation. Fluorescence images were acquired using 3 ms acquisition time, 20% power of a 630 nm LED light source for the Cy5 channel and 5 ms acquisition time, 20% power of a 475 nm LED light source for the mVenus channel. The images were evaluated using FIJI software (63) by automatically adjusting the brightness of the DIC and fluorescence images.

For fluorescence recovery after photobleaching (FRAP) experiments, sample chambers were assembled by lining two stripes of double-sided tape on a glass slide and covering it with a cover slide to form the chamber.

Samples were prepared as described above for the turbidity measurements, with 90% unlabeled and 10% of the respective mVenus-labeled proteins. FRAP experiments were performed on a Leica SP5 confocal microscope using a 488 nm laser and 63× objective at 1% laser power for imaging. The sample was allowed to equilibrate inside the chamber for 15 min after mixing, and the regions of interest were selected for full-bleaching of the condensates. Image size was 41.33 × 41.33 μm with 128 × 128 pixels to enable fast frame rates of 0.06 s. Regions of interest (ROIs) were defined in the software to mark individual condensate areas for bleaching. The time series was set to 30 s of prebleach acquisition, followed by 100% laser power bleach pulses for 10 frames (0.6 s) and 10 min of postbleach acquisition. The FRAP experiments were evaluated using FIJI software (63) by selecting and measuring the mean intensity over time in ROIs for the background, control condensate, and condensates that were bleached by the bleach pulse. For each experiment, the background signal was subtracted, and the control condensate was used to correct for unwanted photobleaching. Normalized intensities were calculated using the following equation:

$$I_{norm} = \frac{I - I_{min,post}}{I_{max,pre} - I_{min,post}} \text{ with}$$

$$I_{max,\ pre} = max\left(\frac{I * c_{max}}{c} - bg\right),$$
$$I_{min,\ post} = min\left(\frac{I * c_{max}}{c} - bg\right) \text{ and}$$
$$c_{max,\ pre} = max\ (c - bg)$$

where *pre* and *post* refer to pre- and postbleach time points being used for evaluation of extrema, and *bg* and *c* are the mean intensities of ROIs from the background and the control condensate, respectively.

FRAP data were analyzed using ImageJ (64). The normalized fluorescence intensities were fitted with a 2D diffusion model with a fixed boundary condition (65). The resulting recovery curve was described by the equation:

$$\mathrm{I_{norm}} = \mathrm{B} \times \left(1 - e^{-t/\tau}\right)$$

where B is the mobile fraction and τ is the characteristic recovery time constant. The half-time of recovery ($t_{1/2}$), i.e., the time it takes for fluorescence to recover to half its maximum, was then calculated as:

$$t_{1/2} = \ln(2) \times \tau.$$

Assuming a uniform radius of the bleached area (r), the diffusion coefficient D was calculated based on the equation derived by Soumpasis (66), which relates $t_{1/2}$ to D by:

$$D = 0.224 \times \left(r^2 / t_{1/2}\right)$$

Since the bleached area (r^2) was different for different experiments, for each individual experiment the diffusion coefficient was calculated from the fitted recovery time constant. Finally, the average and SD were calculated.

Corse-grained molecular dynamics simulations

All residues were modeled using the CALVADOS-2 force field, where each amino acid is represented as a single bead, and the system is treated with implicit solvent (67). The initial configuration for the simulations was generated using HOOBAS (68). The electrostatic screening parameter in force field was explicitly tuned by varying the inverse Debye length (κ) from 0.04 to 0.3 $Å^{-1}$, corresponding to experimental salt concentrations up to 1000 mM.

Simulations were performed using HOOMD-Blue (69,70) in a slab geometry with dimensions of 3000 × 250 × 250 $Å^3$, containing 216 protein chains. The system was evolved in the canonical (NVT) ensemble for 5 μs using Langevin dynamics. The integration time step was set to $\Delta t = 0.01\ \tau$, τ is the unit of time, corresponding to 10 fs. A total of 5000 snapshots were generated, with the first 500 snapshots discarded to allow for equilibration.

The density profile was extracted using a convolution-based method. In phase-separated systems, where the position of the interface fluctuates along the x axis, each profile was centered by aligning the dense phase at x = 0. This centering was achieved by defining a concentration autocorrelation function and shifting the profiles to maximize their overlap with a reference autocorrelation function, following the method described by Jung and Yethiraj (71).

The centered density profiles were then fitted using a symmetric hyperbolic tangent (tanh) function:

$$\rho(x) = \rho_{dil} + 0.5 * (\rho_{den} - \rho_{dil}) * (tanh((x + T - x_0) / w) - tanh((x - T - x_0) / w))$$

where ρ_{dil} and ρ_{den} are the dilute and dense volume fraction, T is the half-thickness of the protein slab, and w is the interface width. This method performs well for strongly phase-separated systems but becomes less reliable near the critical point, where the density contrast diminishes. As such, a fit was considered valid only if the coefficient of determination (R^2) exceeded 0.70, in which case the system was classified as phase-separated (two-phase) state. Otherwise, the system was identified as mixed (one-phase) state. Exact phase boundaries are therefore subject to noise in the profile. Results were further confirmed by visual inspection.

Simulations were performed for 10 values of the screening parameter κ, covering temperatures from 200 to 235 K for the IM30 system and from 200 to 245 K for the IM30* system. The results are presented in the main text. Simulation data handling, and workflow management were handled by signac and signac-flow (72–74).

In vivo localization of fluorescently labeled IM30

The construction of *Synechocystis* sp. PCC 6803 cells stably expressing mVenus-tagged IM30 upon induction was described recently (75). For analyzes, the *Synechocystis* cultures were maintained in a shaker at 30°C under continuous, low-intensity white light (30 μmol photons m^{-2} s^{-1}) in BG11 medium (76). Before the analyses, the cells were diluted to an optical density of about 1 at 750 nm, and gene expression was induced by adding 0.1 mg/mL L-rhamnose. Following a 1-day incubation period, the cells were visualized with a Zeiss Axio Observer.Z1 microscope (Zeiss, Oberkochen, Germany) equipped with a Zeiss ApoTome.2 to remove out-of-focus light, featuring a 63×/1.4 oil immersion objective. For image acquisition, cells were immobilized on 2% agarose. The mVenus was excited at 450–490 nm and imaged at 500–550 nm. Images were processed using the ZEN software (version 2.3.64.0) supplied by the vendor. Salt-stressed *Synechocystis* cells were imaged upon incubating the IM30-mVenus expressing *Synechocystis* cells for 15 min in BG11 medium containing 0.5 or 1 M NaCl.

RESULTS

NaCl-induced formation of IM30* condensates in crowded environments

Diverse cellular constraints can trigger and modulate the formation of biomolecular condensate in vivo and in vitro, including molecular crowding and ionic strength (77,78). Often, IDPs or proteins containing IDRs are involved in

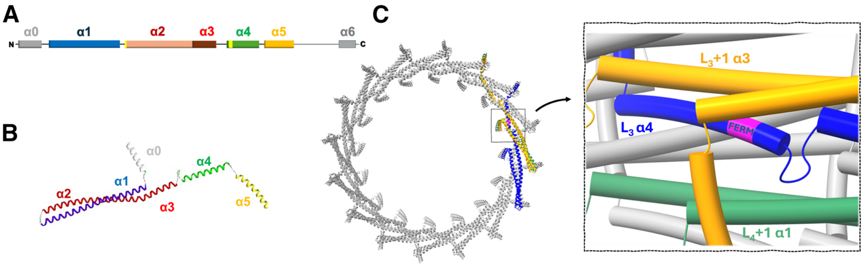

FIGURE 1 The structure of IM30(*). (*A*) Schematic representation of the full-length IM30(*) protein with α-helical regions (α0-α6) indicated. Only in an oligomeric assembly, helices α0 and α4-α6 are structured. In monomeric IM30, these regions are disordered. The position of amino acids mutated in IM30* are indicated by yellow boxes. In (*B*) the structure of monomeric IM30 extracted from an oligomeric barrel assembly (PDB: 7O3Y) is shown. Note that the structures of the regions following helix α5 were not solved. (*C*) Multiple IM30 monomers oligomerize to form planar ring structures (shown). Six to seven of such rings stack to form the barrel structures observed in the cryoelectron microscopy analyses. A conserved interaction between helix α4 of one ring layer with helix α3 of a stacking layer crucially stabilizes the oligomeric assembly. In IM30* the four residues ([168]FERM[171]) are replaced by Ala, which retains the α-helix-forming propensity of this region (44).

phase separation resulting in the formation of condensates (53–55). As a large part of monomeric IM30 is ID (33,44), we aimed at analyzing whether the protein has the potential to phase separate and form biomolecular condensates, which might be linked to the puncta structures observed in vivo, in stressed *Synechocystis* cells.

Under standard (nonstress) growth conditions, IM30 is evenly distributed throughout the cyanobacterial cytoplasm, likely reflecting its monomeric or small oligomeric state. However, the purified protein has a strong propensity to spontaneously assemble into large homo-oligomeric structures in vitro, a process accompanied by the folding of at least helices α0 and α4-α5 (44). While cellular chaperones are known to disassemble larger IM30 oligomers in vivo (79), an engineered variant, IM30*, offers a valuable in vitro model for the monomerized state.

In IM30*, a critical intersubunit interaction is disrupted, preventing the formation of large oligomers (33,44,80) (Fig. 1C). Importantly, the intrinsic helical propensity of individual regions within the oligomeric structure remains unchanged by the mutations, confirming that IM30* effectively recapitulates the disassembled, monomeric state of IM30 (44).

To investigate whether the IDR-containing protein IM30 phase separates and forms condensates under physiologically relevant conditions, particularly in relation to the puncta formation observed in vivo (46,47,51), we first analyzed phase separation of IM30* at physiological ionic strength (100 mM NaCl) and a pH of 7.6, the cytoplasmic pH of cyanobacteria measured when cells were grown at neutral pH (81). However, under these conditions, IM30* did not phase separate (Fig. 2A). Yet, the cytoplasm of a cell is a highly crowded environment filled with macromolecules that occupy 20–30% of the cell volume (82). Crowding has been recognized as an important factor crucial for the formation of biomolecular condensates (83,84), and to mimic the crowding conditions observed within a cell, in vitro studies typically utilize macromolecular crowding agents, such as polyethylene glycol (PEG). In fact, in the presence of 10% PEG as crowding agent, IM30* formed condensates already at low ionic strength (≥25 mM NaCl, Fig. 2, *A* and *B*). Our turbidity data collected in the presence of 10% PEG, 100 mM NaCl, and with increasing protein concentrations suggest a saturation concentration in the lower μM range for NaCl-induced phase separation (Fig. 2, *C* and *D*). While we observed condensate formation already at concentrations below 10% (Fig. S1), 10% PEG appears to provide a crowding level that gives measurable effects for several phase-separating proteins even without completely mimicking the dense cellular interior (83). Thus, we used 10% PEG in our experiments to allow for standardized comparisons across different proteins.

The phase diagram assembled from multiple measurements (Fig. 2 *D*) is reminiscent of a middle salt phase separation (85). Here, the term middle salt does not refer to a defined salt concentration, but rather to a nonmonotonic dependence of phase separation on the salt concentration, where condensate formation is observed only within an intermediate salt regime and suppressed at both low and high ionic strengths. At salt concentrations below 10 mM, no increase in turbidity was observed at any tested protein concentration, suggesting that a minimal concentration of 10–25 mM NaCl is required to induce condensates (Fig. 2, *B* and *D*). Increasing the NaCl concentration above this critical concentration first resulted in condensate formation, whereas further increasing the salt concentration resulted in disassembly of these structures, as observed above 100 mM at 4 and 8 μM IM30* (Fig. 2, *B* and *D*). Thus, there are both a lower and an upper critical NaCl concentration for condensate formation, typical for middle salt phase separation, as further discussed below.

In summary, the formation of IM30* condensates appears to depend on a combination of (at least) the presence of NaCl and crowding. IM30* forms condensates in vitro at physiologically relevant salt concentrations, and deviations from near-physiological concentrations negatively affect the propensity for condensate formation.

Coarse-grained simulations recapitulate the experimentally determined phase diagram

To elucidate the origin of the observed phase separation, we next turned to coarse-grained molecular dynamics (CG MD) simulations. Specifically, we used the CALVADOS force

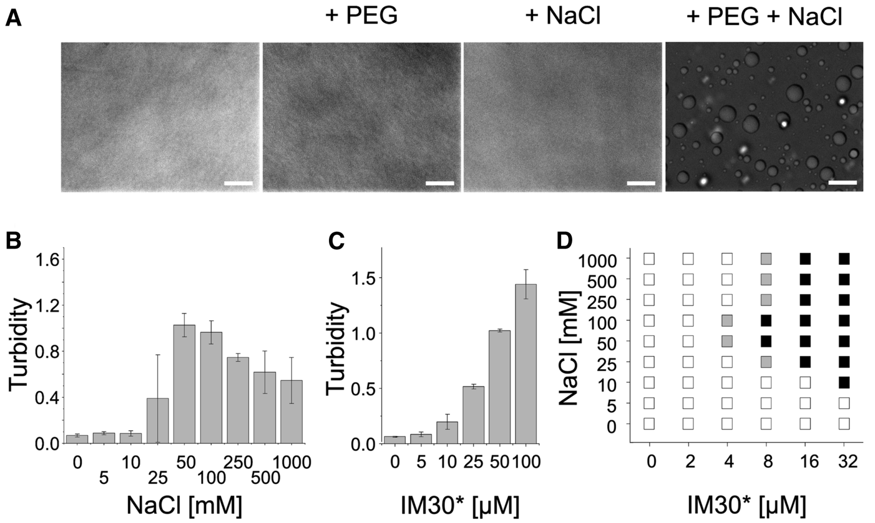

FIGURE 2 Salt-induced in vitro formation of IM30* condensates. (*A*) DIC microscopy images of 32 μM IM30* in the absence or presence of 100 mM NaCl and 10% PEG. Only when both 100 mM NaCl and 10% PEG were present, condensate formation was observed. Scale bars, 10 μm. (*B*) Turbidity of an 8 μM IM30* solution measured in the presence of 10% PEG and increasing NaCl concentrations. (*C*) Turbidity of a solution with a constant NaCl concentration of 100 mM and 10% PEG determined at increasing IM30* concentrations. Error bars in (*B*) and (*C*) represent SD (n = 3 biological replicates). (*D*) Phase diagram of IM30* at various NaCl and protein concentrations in the presence of 10% PEG. All measurements were performed using 20 mM HEPES buffer (pH 7.6). At a turbidity above 0.13, condensates became observable via DIC; thus, this value was set as the lower limit for condensate formation (*gray*). Values ≥0.26 were defined as significant condensate formation (*black*).

field (67) in a slab geometry, and an overall protein concentration of 50 μM. Charge interactions are treated through screened electrostatics, which allows us to tune the salt concentration by varying the Debye length. With an average (mean-field) hydrophobicity of $<\chi> = 0.38$, the protein is considered to be in a favorable solvent environment at 300 K. Consequently, we did not observe phase separation, in line with the experiments.

In experiments, molecular crowders such as PEG promote protein condensation by reducing the availability of solvent, which makes it effectively less favorable for polymers to remain dissolved. While PEG can influence electrostatic screening in real systems, its dominant physical effect, macromolecular crowding, is primarily entropic and equivalent to worsening the solvent quality. In CG simulations of polymer systems, the solvent quality is typically tuned via a single effective parameter, most commonly the temperature, which controls the balance between energetic (attractive) and entropic (dispersive) forces. A lower temperature here corresponds to poorer solvent conditions enhancing self-association, even in the absence of explicit crowders. Thus, we model the effect of PEG by reducing the simulation temperature.

For IM30*, phase separation was observed at 240 K and below (Fig. 3 *B*), whereas for (monomeric) IM30 WT it occurred at 230 K and below (Fig. 3 *C*). This temperature is slightly below the θ temperature, i.e., the condition under which the solvent is just sufficient to maintain the polymer chain in an ideal, unperturbed conformation, corresponding to a scaled interaction strength of $\tau N^{1/2} \approx -2.4$. At this point, the solvent quality is only marginally poor, meaning that hydrophobic interactions alone are too weak to drive phase separation (see Fig. S2). Phase separation is only observed at intermediate salt concentrations, as in the experiments, indicating that the polyampholyte character of IM30 is crucial. Noteworthy, the phase diagrams for IM30 WT and IM30* are highly similar, suggesting that the overall phase separation propensity is not strongly affected by the mutations.

The observed phase behavior aligns well with established theoretical frameworks for polyampholytes, where quasi-neutral polyampholytes can undergo phase separation due to attractive electrostatic interactions between oppositely charged segments, especially when the net charge is low but the total charge density is high (62). Thus, the phase behavior of polyampholyte solutions depends critically on the balance between electrostatic charge correlations arising from the spatial arrangement and interactions of charged groups and entropic contributions from chain conformations and counterion release, as also quantified by recent experimentally validated predictors of phase separation, which quantify how the spatial arrangement of charges along a polyampholyte chain influences the phase separation propensity (86,87). If the temperature is further reduced to 210 K in our simulations, hydrophobic interactions are sufficient to drive phase separation on their own, with $\tau N^{1/2} \sim -4.2$, if a minimal salt concentration is provided to shield the polyelectrolyte nature of IM30*. Consequently, the transition ceases to be re-entrant.

Nature and dynamics of IM30* condensates

Next, we studied the dynamic features of the formed IM30* condensates via following the FRAP of single condensates. IM30* condensates were formed and characterized at pH 7.6 in the presence of 100 mM NaCl and 10% PEG. In the FRAP experiments (Fig. 4, *A* and *B*), the IM30* condensates showed an incomplete recovery after 10 min, yet, the condensates were still able to recover approximately 60% of their fluorescence signal (Fig. 4 *B*) and partially wet the bottom glass surface (Fig. 4 *C*). From the recovery curves (Fig. 4 *B*) we were able to calculate the time it took for

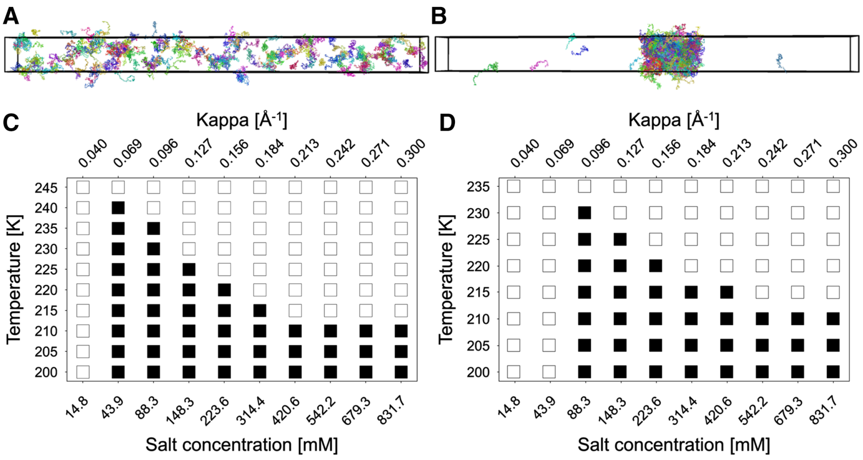

FIGURE 3 Phase separation of IM30* and IM30 WT analyzed via CG molecular dynamics simulations. (*A* and *B*) Snapshots of a simulation in slab configuration in the (*A*) 1-phase regime and (*B*) 2-phase regime. (*C* and *D*) Phase diagrams of (*C*) IM30* and (*D*) IM30 WT extracted from fitting density profiles to the simulation (see methods). Conditions, where two phases coexist, are marked in black. Phase boundaries in (*C*) and (*D*) are subject to uncertainties, as discussed in the methods.

the fluorescence intensity to recover to half of its final value, i.e., the recovery half-time ($t_{1/2}$), which was approximately 96.1 ± 43.5 s, as well as a diffusion coefficient D of 0.0089 ± 0.0029 $\mu m^2/s$.

Upon an initial formation of liquid spherical condensates, such structures transition to more gel-like assemblies in some cases (83,88,89). While some condensates undergo this transition as part of their physiological function, in other cases, changes in fluidity can indicate impaired function, finally leading to the formation of toxic aggregates (90–93). The here observed reduced mobile fraction inside the IM30* condensates suggests that the NaCl/PEG-induced IM30* condensates rapidly transitioned toward a less-mobile state.

In experiments, molecular crowders such as PEG are used to promote phase separation not via a direct chemical interaction but by reducing the availability of the solvent, which makes it less favorable for proteins to remain dissolved. Yet, while PEG is a crowding agent commonly used for studying in vitro condensate formation, the crowder itself has been shown to induce phase separation of proteins in some cases (83,94–96). In fact, it is possible that crowding agents specifically interact with proteins instead of providing a neutral environment that supports condensate formation. If PEG interacts with the protein, we would expect the crowder to colocalize with the protein within the formed condensates. In contrast, if condensate formation displaces the crowder, it is expected to localize preferentially in the solution. Favored incorporation, exclusion, or neutral partitioning of crowding agents can be tested via fluorescence microscopy using a fluorescently labeled crowder (94,97). We here used Cy5-labeled PEG to check preferential partitioning of PEG into condensates. As a control, we first examined two distinct systems undergoing phase separation (Fig. 5, *C–F*): in the PEG/dextran system, dextran-rich condensates preferentially incorporate PEG due to attractive interactions, whereas, in the PEG/BSA system, PEG is excluded from BSA-rich condensates, due to repulsive interactions. In contrast, PEG/NaCl-induced IM30* condensates containing 10% PEG did not show pronounced crowder incorporation or exclusion through attractive or repulsive interactions between IM30 and PEG, respectively (Fig. 5, *A* and *B*). This supports the suitability of PEG as a neutral crowding agent in our experiments.

Consequently, condensate formation induced by the addition of NaCl plus PEG was triggered by the crowded environment rather than by direct chemical interactions of IM30* with PEG, in line with the simulations.

Formation of IM30 condensates requires barrel disassembly

In contrast to IM30*, IM30 WT monomers establish multiple intermolecular contacts in solution resulting in the formation of large homo-oligomeric barrel structures (12,20,21). Furthermore, individual IM30 barrels can stick to each other, stack, and even form extended rods (12,34,37,98,99). The large size of IM30 barrel and/or rod structures inherently causes an increased light scattering, which explains the significant difference in (baseline) turbidity observed between IM30 and IM30* at pH 7.6 (Fig. 6 *B*). When comparing the turbidity of the IM30 and IM30* solutions in the absence vs. presence of NaCl/PEG, we observed a significant increase in turbidity for both, indicating the formation or larger assemblies (Fig. 6 *B*). Consequently, larger structures formed when protein-protein interactions were promoted by crowding conditions, in particular if the protein solubility was reduced by lowering repulsive electrostatic interactions at intermediate ionic strengths (Fig. 6 *B*). However, turbidity measurements alone cannot distinguish between the formation of condensates, i.e., reversible, fluid-like, spherical droplets formed via liquid-liquid phase separation, and the formation of aggregates, i.e., irreversible, solid-like assemblies typically arising from protein misfolding and aberrant interactions of damaged or misfolded proteins.

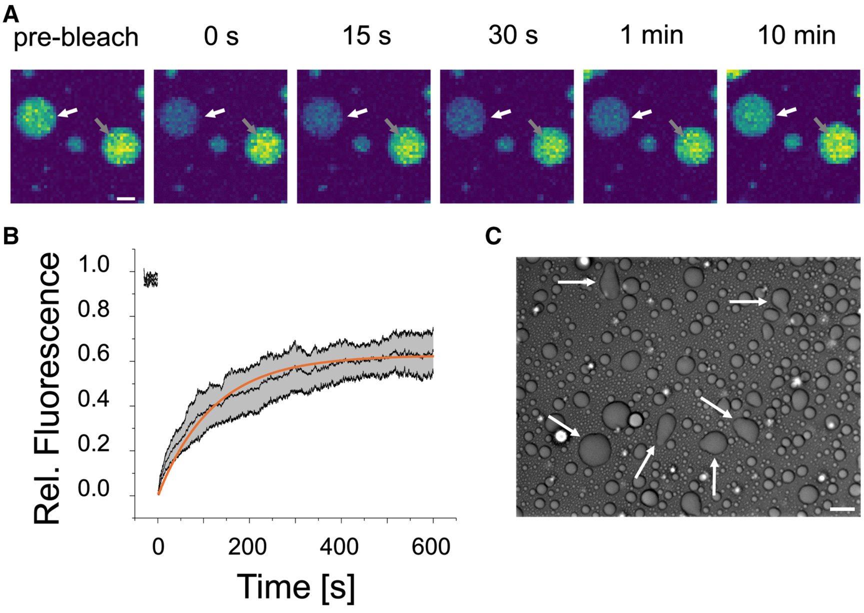

FIGURE 4 Structure and dynamics of IM30* condensates. (*A*) FRAP experiments: fluorescence microscopy images of a fully bleached (*white arrow*) and a nonbleached (*gray arrow*) NaCl/PEG-induced IM30* condensate before and after the bleach pulse. Nonbleached condensates were used as controls to correct for acquisition bleaching. Scale bar: 2 μm. (*B*) Normalized fluorescence intensity measured in FRAP experiments with IM30* condensates. Error bars represent SD ($n = 3$ biological replicates [$N = 6$ condensates]). The red line represents a fit to the normalized average values. From similar fits to the individual FRAP curves the $t_{1/2}$ and D values were determined. (*C*) Wetting of IM30* condensates on glass surfaces. Exemplary DIC image showing wetting of bottom glass surface by IM30* condensates after prolonged incubation time. White arrows indicate some condensates that attached to the glass slide and wet the surface. Condensates were formed in 10 mM HEPES at pH 7.6, in the presence of 100 mM NaCl and 10% PEG. Scale bar: 10 μm.

Yet, these structures are distinguishable via DIC microscopy (Fig. 6 *A*). In fact, while IM30* formed spherical condensates in the presence of NaCl and PEG, the WT protein formed large irregular aggregates.

To determine whether the homo-oligomeric IM30 barrel structure hinders the formation of condensates, we next destabilized IM30 barrels by adding 3 M urea and compared condensate formation of IM30 and IM30* under otherwise identical conditions (Fig. 6, *A* and *B*). At this urea concentration, homo-oligomeric IM30 supercomplexes are largely disassembled exposing the unstructured region of IM30 while the α1-α3 helical hairpin region remains structured (Fig. S3) (44). In the sole presence of 3 M urea, IM30 did not form higher-ordered oligomers, aggregates, or condensates, visible as a decrease in turbidity (Fig. 6, *A* and *B*), in line with recent observations (44). However, when NaCl plus PEG were added to urea-destabilized proteins, the formation of larger assemblies was observed for both IM30 WT and IM30*, visible as an increase in turbidity (Fig. 5 *B*). When these assemblies were analyzed via DIC microscopy, the formation of condensates was confirmed for both proteins (Fig. 6 *A*). Thus, the structure of the IM30 barrels indeed appears to inhibit the formation of condensates and triggers the formation of aggregates under conditions where IM30* readily forms condensates (Fig. 2).

However, while addition of urea promotes condensate formation in the case of IM30 WT, for IM30* a different effect was observed (Fig. 6 *B*): the extent of condensate formation was reduced by 3 M urea (Fig. 6, *A* and *B*), visible as a decreased turbidity compared with the NaCl/PEG-only sample. Thus, urea dissolved the IM30 WT aggregates, which formed in the absence of urea, resulting in partial monomer unfolding (44) and thereby enabling the formation of biomolecular condensates when PEG and NaCl were present (Fig. 6, *A* and *B*). However, condensates formed by IM30* are destabilized by urea. As IM30 and IM30* formed condensates under conditions where IM30 no longer assembled into large homo-oligomers, the interactions driving condensate formation are likely identical for IM30 and IM30*.

Taken together, our data clearly show that the formation of IM30 condensates depends on a combination of NaCl, crowding, plus the oligomeric state (and potentially other factors).

In vivo formation of IM30 puncta in salt-stressed *Synechocystis* cells

The formation of puncta has previously been observed when living *Synechocystis* cells were light stressed (47). Here, we now demonstrate that IM30 can undergo phase separation and forms biomolecular condensates in a crowded environment at moderately elevated NaCl concentrations. To test whether IM30 also assembles into puncta in vivo in salt-stressed *Synechocystis* cells, we next examined IM30-mVenus-expressing cells exposed to an increased extracellular NaCl concentration.

In standard BG11 growth medium, the intracellular Na^+ concentration is ~30 mM (100,101). When the external NaCl concentration is raised, intracellular Na^+ can rise to roughly 10–20% of the external level. For example, supplementation with 684 mM NaCl yields an intracellular Na^+ concentration of ~215 mM in *Synechocystis* (100,101). Accordingly, we incubated IM30-mVenus-expressing *Synechocystis* cells in BG11 supplemented with 0.5 M or 1 M NaCl, respectively, for 15 min and subsequently visualized

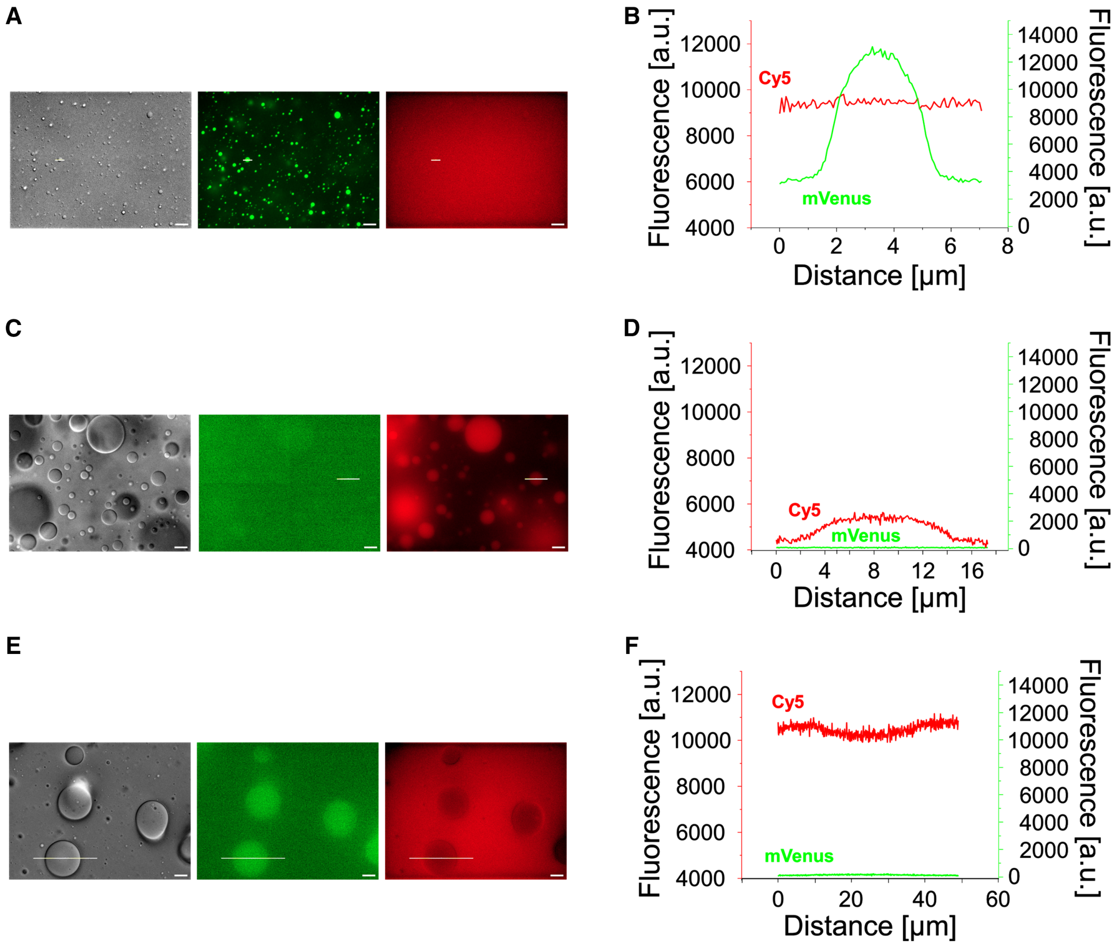

FIGURE 5 Fluorescence microscopy of IM30* condensates in the presence of a fluorescently labeled crowding agent. (*A* and *B*) 32 μM IM30*/IM30*-mVenus (9:1), 10% PEG, 1 mol% Cy5-PEG, 100 mM NaCl, and 20 mM HEPES (pH 7.6). (*C* and *D*) PEG/dextran condensates (control) and (*E* and *F*) PEG/BSA condensates (control) each with the same total concentration of Cy5-PEG as in (*A*) and (*B*). (*A*, *C*, and *E*) DIC image (*left*); mVenus channel (*middle*), and Cy5 channel (*right*). (*B*, *D*, and *F*) Fluorescence signal profiles along the white lines shown in the corresponding images; exemplary images and line profile are shown for a set of $n = 3$ experiments. Scale bars, 10 μm. While in the PEG/dextran system (*C* and *D*), PEG is specifically concentrated in condensates, the crowder is excluded in case of condensates forming in the PEG/BSA system (*E* and *F*).

the cells by fluorescence microscopy (Fig. 7). Under control conditions (no added NaCl), IM30 was uniformly distributed throughout the cyanobacterial cytoplasm. At 0.5 M NaCl, occasional discrete puncta became apparent, and at 1 M NaCl robust puncta formation was clearly evident. Thus, in addition to light stress, salt stress, typically accompanied by osmotic stress, can also induce the formation of intracellular IM30 puncta in living *Synechocystis* cells.

DISCUSSION

IM30 forms condensates in vitro at physiologically relevant conditions

IM30 is localized in the cyanobacterial cytoplasm or chloroplast stroma, respectively, where it is either soluble or attached to internal membranes (34,47,51,102). In monomeric IM30, about 50% of the protein is intrinsically disordered (44), and we here analyzed whether this highly disordered protein is capable of condensate formation, as often observed for IDPs or proteins containing IDRs. The pH within the cytoplasm of cyanobacteria has been estimated to be around 7.6, when the cells are grown at neutral pH (81), and because of this all of our analyses were performed at this pH. When the crowded environment of the cell was mimicked by the addition of 10% PEG, we observed the formation of condensates at physiologically relevant NaCl concentrations when IM30*, the monomeric IM30 variant, was analyzed (Fig. 2).

However, in vitro, IM30 WT proteins tend to cluster and form large aggregates instead of forming liquid-like condensates under these conditions (Fig. 6), which seems to contradict the assumption that the protein can and does form condensates. The observation that IM30*, which cannot form homo-oligomeric barrels anymore, readily forms condensates, indicated that the differences observed

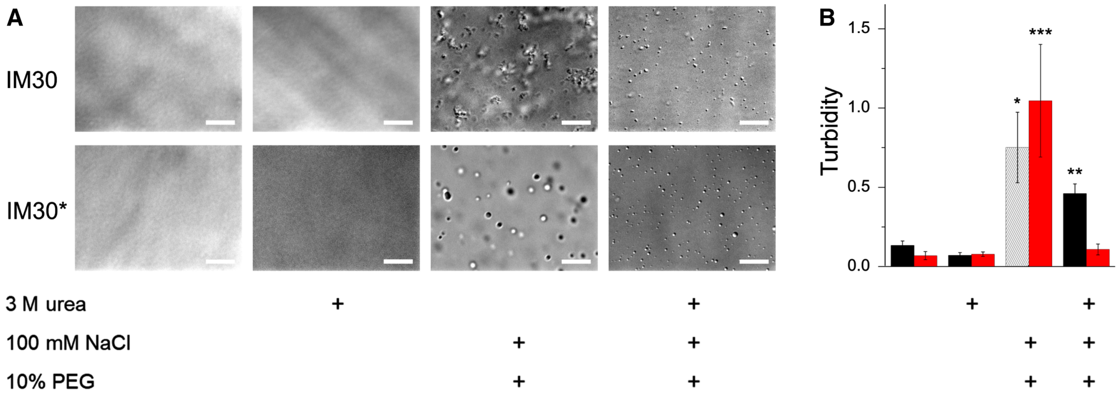

FIGURE 6 Formation of IM30 WT and IM30* aggregates/condensates. (*A*) DIC images of 32 μM IM30 WT or IM30* in the absence or presence of 10% PEG, 100 mM NaCl, and/or 3 M urea, as indicated. Scale bars, 10 μm. (*B*) Column diagram showing the turbidity of solutions containing 32 μM IM30 (*black*) or IM30* (*red*) monitored at varying conditions. Experiments were performed in phosphate buffer (pH 7.6) and changes in the buffer conditions are indicated below the columns. All samples were analyzed via DIC microscopy at each specified condition to distinguish between the formation of aggregates and condensates. Columns in (*B*) with diagonal stripes indicate the formation of large, ill-defined aggregates, in contrast to the formation of defined spherical condensates. Aggregation was observed solely with the IM30 WT protein. Representative images and error bars representing standard deviations were obtained from at least three biological replicates. Statistically significant increases in turbidity, calculated via a Welch's *t* test, are indicated with an asterisk, with significance values: *$p < 0.05$, **$p < 0.01$, ***$p < 0.001$.

are related to the oligomeric state of IM30. In fact, urea-destabilized IM30 WT does phase separate and forms condensates as IM30* (Fig. 6). The assumption that monomeric IM30 WT behaves essentially like (monomeric) IM30* is further supported by the simulations (Fig. 3), which show that the intrinsic propensity to form condensates is not altered due to the mutations. Thus, disassembly of IM30 oligomers in a cellular environment, e.g., mediated by chaperones, could well enable the formation of IM30 condensates. In fact, interaction of chloroplast and cyanobacterial IM30 with Hsp70 chaperones has been shown (47,79) as well as Hsp70-mediated disassembly of large IM30 oligomers (79).

Generally, a protein concentration exceeding a critical saturation concentration c_{sat} is required for condensate formation (103,104). Our experimentally determined phase diagrams indicate a c_{sat} of ~4 μM at a physiological relevant NaCl concentration (Fig. 2D). While the exact c_{sat} value is modulated both in vitro and in vivo by various factors, including temperature, ionic strength, and posttranslational modifications, this value serves here as a reference point.

Based on a study investigating the in vivo abundance of several proteins in *Synechocystis*, IM30 is present at 40,500–45,000 copies per cell (105). Assuming that a *Synechocystis* cell has an average diameter *d* of 1–2 μm and a spherical shape, the IM30 concentration inside a single *Synechocystis* cell is 128–143 μM for $d = 1$ μm and 16–18 μM for $d = 2$ μm. The estimated concentration represents a lower limit, because a significant portion of the cell volume is occupied by the TM system. Therefore, the estimated in vivo concentration is significantly higher than the saturation concentration of approximately 4 μM determined in our in vitro analyses with IM30* (Fig. 3, *D* and *H*) and is perfectly in line with the physiologically relevant range for the proposed in vivo formation of IM30 condensates. Noteworthy, even at concentrations as high as 140 μM, IM30 still forms condensates, and condensate formation still requires the presence of a crowded environment (Fig. S4).

Thus, IM30 forms condensates at physiologically relevant NaCl and protein concentrations, and one can only wonder why the protein does not always form condensates in vivo. Likely, oligomer formation and/or interaction of IM30 monomers with other factors hinder the formation of condensates in unstressed cells.

The nature and characteristics of IM30 condensates

Our experimental and simulation results (Figs. 2 and 3) clearly show that a lower critical salt concentration exists at 10–25 mM NaCl and that IM30* condensate formation is mostly enhanced at a physiologically relevant concentration of 100 mM NaCl. Since condensates still formed at NaCl concentrations of 500 and 1000 mM (Fig. 2), it is likely that the interactions driving condensate formation are not entirely electrostatic in nature. If they were, we would expect these high NaCl concentrations to have a significantly stronger inhibitory effect on condensate formation, as observed with other proteins (78,106). In fact, the experimentally determined IM30* phase separation is characterized by a re-entrant behavior, which likely arises from the competing effects of electrostatic screening: at low salt, long-range repulsions prevent coalescence, whereas at high salt, excessive screening disrupts attractive electrostatic interactions necessary for phase separation. Thus, phase separation peaks in a "middle" window of salt concentration, reflecting a balance between electrostatic screening (promoting re-entrance) and

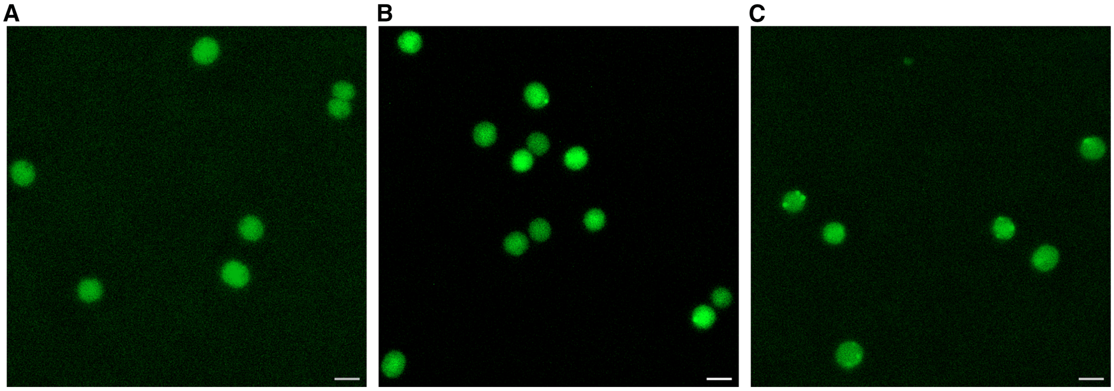

FIGURE 7 IM30 *puncta* formation in salt-stressed *Synechocystis* cells. *Synechocystis* cells expressing mVenus-tagged IM30 were cultivated in (*A*) standard growth medium or (*B* and *C*) salt-stressed for 15 min. with medium containing (*B*) 0.5 M or (*C*) 1 M NaCl. Formation of *puncta* structures was already observed at 0.5 M NaCl, yet, was clearly more pronounced at 1 M NaCl. Scale bars = 2 μm.

salting-out (opposing it). Our experimentally determined phase diagram likely shows an overlap of these two effects, as they correspond well to the area around 100 mM NaCl with the lowest saturation concentrations and the area at higher ionic strength with increased saturation concentration. This assumption is also supported by our simulations, where re-entrant phase separation was observed, e.g., at 220 K but not at 210 K because lower temperature mimics poorer solvent quality, favoring interactions even at high salt. The simulation temperature here is an effective parameter representing the solvent quality, and lowering the temperature mimics the addition of crowding agents or salting-out ions in experiments. Of note, our CG simulations do not consider salting-out effects, which further reduce solvent quality at high salt and can suppress re-entrance. The agreement of our simulation results and the experimental observations at intermediate salt concentrations suggests that our simulations capture the dominant electrostatic contribution, despite simplifying other effects.

Implications of condensate formation for the IM30 in vivo function

As shown recently and here, the formation of membrane-covering IM30 carpets, membrane internalization into IM30 rings and/or rods, as well as condensate formation require the disassembly of (preexisting) large IM30 homo-oligomeric structures (12,33). In fact, the formation of higher-order IM30 assemblies modulates membrane binding (80) as well as condensate formation (Fig. 6). Yet, interaction of IM30 with Hsp70 chaperones has been shown for the proteins of *Synechocystis* and *Chlamydomonas reinhardtii* (47,79), and thus the activity of Hsp70, and potentially other chaperones, likely controls the monomer-oligomer equilibrium in vivo (79). It is expected that IM30 monomers, ordered oligomers, and condensates coexist and are physiologically relevant in vivo, and that their respective concentrations are regulated by interacting proteins.

Biomolecular condensates often emerge in response to stress conditions, and may play a role in protecting cells under stress (107,108). In fact, IM30 forms puncta in vivo with a higher frequency in response to environmental stresses that affect the stability of internal membranes (46,47,51). We now show that the in vivo formation of IM30 puncta, which potentially represent condensates, can also be induced by salt stress (Fig. 7). The basal *Synechocystis* BG11 growth medium contains approximately 20 mM NaCl. However, analyses across various cyanobacterial species have indicated that internal Na^+ concentrations can reach 10–20% of the external levels (100). For example, when *Synechocystis* cells were transferred from standard BG11 medium to media supplemented with 342 or 684 mM NaCl, the intracellular Na^+ concentrations rose from ~30 to ~70 and ~215 mM, respectively (100,101). This salt-dependent increase in the intracellular ionic strength likely promotes the formation of IM30 condensates, suggesting that the now in vitro observed phase separation could serve as a regulatory mechanism for the in vivo IM30 activity. In this context, IM30 may function as a tunable sensor or responder to ionic perturbations, typically coupled with osmotic stress. As IM30, a protein involved in membrane remodeling, can form condensates under physiologically relevant conditions (this study) and puncta structures are observed in vivo under membrane stress conditions, it is reasonable to assume a connection between membrane stabilization/repair and IM30 condensate formation. The formation of IM30 condensates could facilitate the localized and transient accumulation of multiple IM30 monomers in an unassembled state. This process is energetically beneficial, as it circumvents the energy expenditure typically required to disassemble IM30 oligomers into their monomeric form, necessary for the subsequent formation of membrane-associated structures, such as surface-covering (e.g., carpets, spirals) or membrane-internalizing, oligomeric structures (e.g., barrels, rods) that have been observed previously (12,13,20–22,31–37). Nevertheless, the suggested connection between IM30 condensates and membrane

interaction and remodeling, as well as their broader physiological significance, remains incompletely understood and requires further investigation.

DATA AND CODE AVAILABILITY

Any additional information required to reanalyze the data reported in this paper is available from the lead contact upon request.

ACKNOWLEDGMENTS

This work was funded by the Max-Planck Graduate Center at the Max Planck institutes and the University of Mainz, as well as by the Deutsche Forschungsgemeinschaft (DFG, SCHN 690/16-1 to D.S. and SFB1551 (project no. 464588647) to M.G. and D.S.). We thank Mirka Kutzner for assisting in preparing Fig. 1.

AUTHOR CONTRIBUTIONS

Conceptualization, N.Q., N.H., and D.S.; data curation, N.Q., K.D., and N.H.; formal analysis, N.Q., T.R.B., K.D., N.H., M.G., and D.S.; investigation (molecular biology, protein expression and purification, turbidity and FRAP measurements, DIC micrcoscopy, and low-resolution fluorescence microscopy), N.Q.; investigation (CG simulations), T.R.B.; investigation (protein expression and purification, turbidity measurements, DIC and low-resolution fluorescence microscopy), K.D.; methodology, N.Q., T.R.B., K.D., M.G., and D.S.; validation, N.Q., T.R.B., K.D., and N.H.; visualization, N.Q., T.R.B., K.D., and N.H.; writing – original draft, N.Q., T.R.B., M.G., and D.S.; writing – review & editing, K.D., N.H., M.G., and D.S.; funding acquisition, M.G. and D.S.; project administration, M.G. and D.S.; supervision, M.G. and D.S.; resources, D.S.

DECLARATION OF INTERESTS

The authors declare no competing interests.

SUPPORTING MATERIAL

Supporting material can be found online at https://doi.org/10.1016/j.bpj.2026.01.011.

REFERENCES

1. Kroll, D., K. Meierhoff, …, P. Westhoff. 2001. VIPP1, a nuclear gene of Arabidopsis thaliana essential for thylakoid membrane formation. *Proc. Natl. Acad. Sci. USA.* 98:4238–4242.
2. Westphal, S., L. Heins, …, U. C. Vothknecht. 2001. Vipp1 deletion mutant of Synechocystis: a connection between bacterial phage shock and thylakoid biogenesis? *Proc. Natl. Acad. Sci. USA.* 98:4243–4248.
3. Vothknecht, U. C., S. Otters, …, D. Schneider. 2012. Vipp1: a very important protein in plastids? *J. Exp. Bot.* 63:1699–1712.
4. Johnson, M. P. 2025. Structure, regulation and assembly of the photosynthetic electron transport chain. *Nat. Rev. Mol. Cell Biol.* 26:667–690.
5. Pribil, M., M. Labs, and D. Leister. 2014. Structure and dynamics of thylakoids in land plants. *J. Exp. Bot.* 65:1955–1972.
6. Gould, S. B., R. F. Waller, and G. I. McFadden. 2008. Plastid evolution. *Annu. Rev. Plant Biol.* 59:491–517.
7. Ponce-Toledo, R. I., P. Deschamps, …, D. Moreira. 2017. An Early-Branching Freshwater Cyanobacterium at the Origin of Plastids. *Curr. Biol.* 27:386–391.
8. Popp, P. F., V. M. Gumerov, …, D. Wolf. 2022. Phyletic Distribution and Diversification of the Phage Shock Protein Stress Response System in Bacteria and Archaea. *mSystems.* 7:e0134821.
9. Ravi, J., V. Anantharaman, …, M. L. Gennaro. 2024. The phage shock protein (PSP) envelope stress response: discovery of novel partners and evolutionary history. *mSystems.* 9:e0084723.
10. Williams, T. A., and H. H. Low. 2025. The evolution and mechanism of bacterial and archaeal ESCRT-III-like systems. *Curr. Opin. Struct. Biol.* 93:103111.
11. McCullough, J., and W. I. Sundquist. 2025. Thylakoid membrane remodeling by VIPP1 ESCRT-III-like filaments. *Nat. Struct. Mol. Biol.* 32:414–417.
12. Junglas, B., D. Kartte, …, C. Sachse. 2025. Structural basis for Vipp1 membrane binding: from loose coats and carpets to ring and rod assemblies. *Nat. Struct. Mol. Biol.* 32:555–570.
13. Siebenaller, C., B. Junglas, and D. Schneider. 2019. Functional Implications of Multiple IM30 Oligomeric States. *Front. Plant Sci.* 10:1500.
14. Junglas, B., and D. Schneider. 2018. What is Vipp1 good for? *Mol. Microbiol.* 108:1–5.
15. Heidrich, J., A. Thurotte, and D. Schneider. 2017. Specific interaction of IM30/Vipp1 with cyanobacterial and chloroplast membranes results in membrane remodeling and eventually in membrane fusion. *Biochim. Biophys. Acta. Biomembr.* 1859:537–549.
16. Zhang, L., and W. Sakamoto. 2013. Possible function of VIPP1 in thylakoids: Protection but not formation? *Plant Signal. Behav.* 8:e22860.
17. Zhang, L., M. Kusaba, …, W. Sakamoto. 2016. Protection of Chloroplast Membranes by VIPP1 Rescues Aberrant Seedling Development in Arabidopsis nyc1 Mutant. *Front. Plant Sci.* 7:533.
18. Zhang, L., Y. Kato, …, W. Sakamoto. 2012. Essential Role of VIPP1 in Chloroplast Envelope Maintenance in Arabidopsis. *Plant Cell.* 24:3695–3707.
19. Junglas, B., S. T. Huber, …, C. Sachse. 2021. PspA adopts an ESCRT-III-like fold and remodels bacterial membranes. *Cell.* 184:3674–3688.e18.
20. Liu, J., M. Tassinari, …, H. H. Low. 2021. Bacterial Vipp1 and PspA are members of the ancient ESCRT-III membrane-remodeling superfamily. *Cell.* 184:3660–3673.e18.
21. Gupta, T. K., S. Klumpe, …, B. D. Engel. 2021. Structural basis for VIPP1 oligomerization and maintenance of thylakoid membrane integrity. *Cell.* 184:3643–3659.e23.
22. Schlösser, L., C. Sachse, …, D. Schneider. 2023. Conserved structures of ESCRT-III superfamily members across domains of life. *Trends Biochem. Sci.* 48:993–1004.
23. Hudina, E., S. Schott-Verdugo, …, C. Sachse. 2025. The bacterial ESCRT-III PspA rods thin lipid tubules and increase membrane curvature through helix α0 interactions. *Proc. Natl. Acad. Sci. USA.* 122:e2506286122.
24. McCullough, J., A. K. Clippinger, …, A. Frost. 2015. Structure and membrane remodeling activity of ESCRT-III helical polymers. *Science.* 350:1548–1551.
25. Hennig, R., A. West, …, D. Schneider. 2017. The IM30/Vipp1 C-terminus associates with the lipid bilayer and modulates membrane fusion. *Biochim. Biophys. Acta. Bioenerg.* 1858:126–136.
26. Zhang, L., H. Kondo, …, W. Sakamoto. 2016. VIPP1 Has a Disordered C-Terminal Tail Necessary for Protecting Photosynthetic Membranes against Stress. *Plant Physiol.* 171:1983–1995.
27. Ma, L., B. Dong, …, W. Sakamoto. 2025. VESICLE-INDUCING PROTEIN IN PLASTIDS 1 from thylakoid-lacking Gloeobacter promotes thylakoid formation in Arabidopsis. *Plant Physiol.* 199:kiaf359.
28. Pfitzner, A. K., J. Moser von Filseck, and A. Roux. 2021. Principles of membrane remodeling by dynamic ESCRT-III polymers. *Trends Cell Biol.* 31:856–868.

29. McCullough, J., and W. I. Sundquist. 2020. Membrane Remodeling: ESCRT-III Filaments as Molecular Garrotes. *Curr. Biol.* 30:R1425–R1428.

30. McCullough, J., A. Frost, and W. I. Sundquist. 2018. Structures, Functions, and Dynamics of ESCRT-III/Vps4 Membrane Remodeling and Fission Complexes. *Annu. Rev. Cell Dev. Biol.* 34:85–109.

31. Naskar, S., A. Merino, …, H. H. Low. 2025. Mechanism for Vipp1 spiral formation, ring biogenesis, and membrane repair. *Nat. Struct. Mol. Biol.* 32:571–584.

32. Pan, S., K. Gries, …, S. Scheuring. 2024. The cyanobacterial protein VIPP1 forms ESCRT-III-like structures on lipid bilayers. *Nat. Struct. Mol. Biol.* 32:543–554.

33. Junglas, B., R. Orru, …, D. Schneider. 2020. IM30 IDPs form a membrane protective carpet upon super-complex disassembly. *Commun. Biol.* 3:595.

34. Fuhrmann, E., J. B. Bultema, …, D. Schneider. 2009. The vesicle-inducing protein 1 from Synechocystis sp. PCC 6803 organizes into diverse higher-ordered ring structures. *Mol. Biol. Cell.* 20:4620–4628.

35. Aseeva, E., F. Ossenbühl, …, U. C. Vothknecht. 2004. Complex formation of Vipp1 depends on its alpha-helical PspA-like domain. *J. Biol. Chem.* 279:35535–35541.

36. Thurotte, A., and D. Schneider. 2019. The Fusion Activity of IM30 Rings Involves Controlled Unmasking of the Fusogenic Core. *Front. Plant Sci.* 10:108.

37. Saur, M., R. Hennig, …, D. Schneider. 2017. A Janus-Faced IM30 Ring Involved in Thylakoid Membrane Fusion Is Assembled from IM30 Tetramers. *Structure.* 25:1380–1390.e5.

38. Nachmias, D., B. P. Frohn, …, N. Elia. 2025. ESCRTs - a multi-purpose membrane remodeling device encoded in all life forms. *Trends Microbiol.* 33:665–687.

39. Vietri, M., M. Radulovic, and H. Stenmark. 2020. The many functions of ESCRTs. *Nat. Rev. Mol. Cell Biol.* 21:25–42.

40. Remec Pavlin, M., and J. H. Hurley. 2020. The ESCRTs - converging on mechanism. *J. Cell Sci.* 133:jcs240333.

41. Tang, S., W. M. Henne, …, S. D. Emr. 2015. Structural basis for activation, assembly and membrane binding of ESCRT-III Snf7 filaments. *eLife.* 4:e12548.

42. Bajorek, M., H. L. Schubert, …, W. I. Sundquist. 2009. Structural basis for ESCRT-III protein autoinhibition. *Nat. Struct. Mol. Biol.* 16:754–762.

43. Lata, S., M. Roessle, …, W. Weissenhorn. 2008. Structural basis for autoinhibition of ESCRT-III CHMP3. *J. Mol. Biol.* 378:818–827.

44. Quarta, N., T. R. Bhandari, …, D. Schneider. 2024. Monomer unfolding of a bacterial ESCRT-III superfamily member is coupled to oligomer disassembly. *Protein Sci.* 33:e5187.

45. Gachie, S. W., A. Muhire, …, W. Sakamoto. 2025. The thylakoid membrane remodeling protein VIPP1 forms bundled oligomers in tobacco chloroplasts. *Plant Physiol.* 198:kiaf137.

46. Gates, C., N. C. Hill, …, J. C. Cameron. 2022. Kinetics and targeting of Vipp1 aggregation in cyanobacteria. Preprint at bioRxiv. https://doi.org/10.1101/2022.12.01.518719.

47. Bryan, S. J., N. J. Burroughs, …, C. W. Mullineaux. 2014. Localisation and interactions of the Vipp1 protein in cyanobacteria. *Mol. Microbiol.* 94:1179–1195.

48. Engl, C., G. Jovanovic, …, M. Buck. 2009. In vivo localizations of membrane stress controllers PspA and PspG in Escherichia coli. *Mol. Microbiol.* 73:382–396.

49. Yamaguchi, S., D. A. Reid, …, A. J. Darwin. 2013. Changes in Psp protein binding partners, localization and behaviour upon activation of the Yersinia enterocolitica phage shock protein response. *Mol. Microbiol.* 87:656–671.

50. Dominguez-Escobar, J., D. Wolf, …, T. Mascher. 2014. Subcellular localization, interactions and dynamics of the phage-shock protein-like Lia response in Bacillus subtilis. *Mol. Microbiol.* 92:716–732.

51. Gutu, A., F. Chang, and E. K. O'Shea. 2018. Dynamical localization of a thylakoid membrane binding protein is required for acquisition of photosynthetic competency. *Mol. Microbiol.* 108:16–31.

52. Thurotte, A., T. Brüser, …, D. Schneider. 2017. Membrane chaperoning by members of the PspA/IM30 protein family. *Commun. Integr. Biol.* 10:e1264546.

53. Pappu, R. V., S. R. Cohen, …, M. Kar. 2023. Phase Transitions of Associative Biomacromolecules. *Chem. Rev.* 123:8945–8987.

54. Zeng, X., and R. V. Pappu. 2023. Developments in describing equilibrium phase transitions of multivalent associative macromolecules. *Curr. Opin. Struct. Biol.* 79:102540.

55. Borcherds, W., A. Bremer, …, T. Mittag. 2021. How do intrinsically disordered protein regions encode a driving force for liquid-liquid phase separation? *Curr. Opin. Struct. Biol.* 67:41–50.

56. Joshi, A., and S. Mukhopadhyay. 2023. Biophysics of biomolecular condensates. *Biophys. J.* 122:737–740.

57. Mittag, T., and R. V. Pappu. 2022. A conceptual framework for understanding phase separation and addressing open questions and challenges. *Mol. Cell.* 82:2201–2214.

58. Murthy, A. C., G. L. Dignon, …, N. L. Fawzi. 2019. Molecular interactions underlying liquid-liquid phase separation of the FUS low-complexity domain. *Nat. Struct. Mol. Biol.* 26:637–648.

59. Joshi, A., A. Walimbe, …, S. Mukhopadhyay. 2023. Single-molecule FRET unmasks structural subpopulations and crucial molecular events during FUS low-complexity domain phase separation. *Nat. Commun.* 14:7331.

60. Dobrynin, A. V., M. Rubinstein, and S. P. Obukhov. 1996. Cascade of Transitions of Polyelectrolytes in Poor Solvents. *Macromolecules.* 29:2974–2979.

61. Lee, N. K., M. K. Chae, …, J. F. Joanny. 2023. Polyelectrolytes: From Seminal Works to the Influence of the Charge Sequence. *Polymers.* 15:4593.

62. Higgs, P. G., and J.-F. Joanny. 1991. Theory of polyampholyte solutions. *J. Chem. Phys.* 94:1543–1554.

63. Schindelin, J., I. Arganda-Carreras, …, A. Cardona. 2012. Fiji: an open-source platform for biological-image analysis. *Nat. Methods.* 9:676–682.

64. Rueden, C. T., J. Schindelin, …, K. W. Eliceiri. 2017. ImageJ2: ImageJ for the next generation of scientific image data. *BMC Bioinf.* 18:529.

65. Taylor, N. O., M. T. Wei, …, C. P. Brangwynne. 2019. Quantifying Dynamics in Phase-Separated Condensates Using Fluorescence Recovery after Photobleaching. *Biophys. J.* 117:1285–1300.

66. Soumpasis, D. M. 1983. Theoretical analysis of fluorescence photobleaching recovery experiments. *Biophys. J.* 41:95–97.

67. Tesei, G., and K. Lindorff-Larsen. 2022. Improved predictions of phase behaviour of intrinsically disordered proteins by tuning the interaction range. *Open Res. Eur.* 2:94.

68. Girard, M., A. Ehlen, …, M. O. de la Cruz. 2019. Hoobas: A highly object-oriented builder for molecular dynamics. *Comput. Mater. Sci.* 167:25–33.

69. Anderson, J. A., J. Glaser, and S. C. Glotzer. 2020. HOOMD-blue: A Python package for high-performance molecular dynamics and hard particle Monte Carlo simulations. *Comput. Mater. Sci.* 173:109363.

70. Howard, M. P., A. Statt, …, A. Z. Panagiotopoulos. 2019. Quantized bounding volume hierarchies for neighbor search in molecular simulations on graphics processing units. *Comput. Mater. Sci.* 164:139–146.

71. Jung, H., and A. Yethiraj. 2018. A simulation method for the phase diagram of complex fluid mixtures. *J. Chem. Phys.* 148:244903.

72. Dice, B. D., B. L. Butler, …, S. C. Glotzer. 2021. signac: Data Management and Workflows for Computational Researchers. *In* 20th Python in Science Conference, pp. 23–32. https://doi.org/10.25080/majora-1b6fd038-003.

73. Ramasubramani, V., C. S. Adorf, …, S. C. Glotzer. 2018. signac: A Python framework for data and workflow management. *In* 17th Python in Science Conference, pp. 152–159. https://doi.org/10.25080/Majora-4af1f417-016.

74. Adorf, C. S., P. M. Dodd, …, S. C. Glotzer. 2018. Simple data and workflow management with the signac framework. *Comput. Mater. Sci.* 146:220–229.

75. Schlösser, L., and M. Kutzner. 2025. Membrane binding of a cyanobacterial ESCRT-III protein crucially involves the helix alpha1-3 hairpin conserved in all superfamily members. *Protein Sci.* 34:e70387.

76. Rippka, R., R. Y. Stanier, …, J. B. Waterbury. 1979. Generic assignments, strains histories and properties of pure cultures of cyanobacteria. *J. Gen. Microbiol.* 111:1–61.

77. Zhang, Y., N. Xu, …, Z. Xu. 2024. Live-Cell Imaging to Resolve Salt-Induced Liquid–Liquid Phase Separation of FUS Protein by Dye Self-Labeling. *Chem. Biomed. Imaging.* 2:70–80.

78. Krainer, G., T. J. Welsh, …, T. P. J. Knowles. 2021. Reentrant liquid condensate phase of proteins is stabilized by hydrophobic and non-ionic interactions. *Nat. Commun.* 12:1085.

79. Liu, C., F. Willmund, …, M. Schroda. 2007. The chloroplast HSP70B-CDJ2-CGE1 chaperones catalyse assembly and disassembly of VIPP1 oligomers in Chlamydomonas. *Plant J.* 50:265–277.

80. Heidrich, J., V. Wulf, …, D. Schneider. 2016. Organization into Higher Ordered Ring Structures Counteracts Membrane Binding of IM30, a Protein Associated with Inner Membranes in Chloroplasts and Cyanobacteria. *J. Biol. Chem.* 291:14954–14962.

81. Peschek, G. A., T. Czerny, …, W. H. Nitschmann. 1985. Transmembrane Proton Electrochemical Gradients in Dark Aerobic and Anaerobic Cells of the Cyanobacterium (Blue-Green Alga) Anacystis nidulans: Evidence for Respiratory Energy Transduction in the Plasma Membrane. *Plant Physiol.* 79:278–284.

82. Ellis, R. J. 2001. Macromolecular crowding: obvious but underappreciated. *Trends Biochem. Sci.* 26:597–604.

83. Andre, A. A. M., and E. Spruijt. 2020. Liquid-Liquid Phase Separation in Crowded Environments. *Int. J. Mol. Sci.* 21:5908.

84. Rivas, G., and A. P. Minton. 2016. Macromolecular Crowding In Vitro, In Vivo, and In Between. *Trends Biochem. Sci.* 41:970–981.

85. Wohl, S., M. Jakubowski, and W. Zheng. 2021. Salt-Dependent Conformational Changes of Intrinsically Disordered Proteins. *J. Phys. Chem. Lett.* 12:6684–6691.

86. Das, R. K., and R. V. Pappu. 2013. Conformations of intrinsically disordered proteins are influenced by linear sequence distributions of oppositely charged residues. *Proc. Natl. Acad. Sci. USA.* 110:13392–13397.

87. Sawle, L., and K. Ghosh. 2015. A theoretical method to compute sequence dependent configurational properties in charged polymers and proteins. *J. Chem. Phys.* 143:085101.

88. Van Lindt, J., A. Bratek-Skicki, …, P. Tompa. 2021. A generic approach to study the kinetics of liquid-liquid phase separation under near-native conditions. *Commun. Biol.* 4:77.

89. Franzmann, T. M., M. Jahnel, …, S. Alberti. 2018. Phase separation of a yeast prion protein promotes cellular fitness. *Science.* 359:eaao5654.

90. Alberti, S., and D. Dormann. 2019. Liquid-Liquid Phase Separation in Disease. *Annu. Rev. Genet.* 53:171–194.

91. Ray, S., N. Singh, …, S. K. Maji. 2020. alpha-Synuclein aggregation nucleates through liquid-liquid phase separation. *Nat. Chem.* 12:705–716.

92. Mathieu, C., R. V. Pappu, and J. P. Taylor. 2020. Beyond aggregation: Pathological phase transitions in neurodegenerative disease. *Science.* 370:56–60.

93. Dormann, D., and E. A. Lemke. 2024. Adding intrinsically disordered proteins to biological ageing clocks. *Nat. Cell Biol.* 26:851–858.

94. Andre, A. A. M., N. A. Yewdall, and E. Spruijt. 2023. Crowding-induced phase separation and gelling by co-condensation of PEG in NPM1-rRNA condensates. *Biophys. J.* 122:397–407.

95. Rayman, J. B., K. A. Karl, and E. R. Kandel. 2018. TIA-1 Self-Multimerization, Phase Separation, and Recruitment into Stress Granules Are Dynamically Regulated by Zn(2). *Cell Rep.* 22:59–71.

96. Wang, Y., M. Sarkar, …, G. J. Pielak. 2012. Macromolecular crowding and protein stability. *J. Am. Chem. Soc.* 134:16614–16618.

97. Marianelli, A. M., B. M. Miller, and C. D. Keating. 2018. Impact of macromolecular crowding on RNA/spermine complex coacervation and oligonucleotide compartmentalization. *Soft Matter.* 14:368–378.

98. Theis, J., T. K. Gupta, …, M. Schroda. 2019. VIPP1 rods engulf membranes containing phosphatidylinositol phosphates. *Sci. Rep.* 9:8725.

99. Heidrich, J., B. Junglas, …, D. Schneider. 2018. Mg(2+) binding triggers rearrangement of the IM30 ring structure, resulting in augmented exposure of hydrophobic surfaces competent for membrane binding. *J. Biol. Chem.* 293:8230–8241.

100. Hagemann, M. 2011. Molecular biology of cyanobacterial salt acclimation. *FEMS Microbiol. Rev.* 35:87–123.

101. Hagemann, M., S. Fulda, and H. Schubert. 1994. DNA, RNA, and protein synthesis in the cyanobacterium Synechocystis sp. PCC 6803 adapted to different salt concentrations. *Curr. Microbiol.* 28:201–207.

102. Li, H. M., Y. Kaneko, and K. Keegstra. 1994. Molecular cloning of a chloroplastic protein associated with both the envelope and thylakoid membranes. *Plant Mol. Biol.* 25:619–632.

103. Hyman, A. A., C. A. Weber, and F. Jülicher. 2014. Liquid-liquid phase separation in biology. *Annu. Rev. Cell Dev. Biol.* 30:39–58.

104. Harmon, T. S., A. S. Holehouse, …, R. V. Pappu. 2017. Intrinsically disordered linkers determine the interplay between phase separation and gelation in multivalent proteins. *eLife.* 6:e30294.

105. Jackson, P. J., A. Hitchcock, …, C. N. Hunter. 2023. Absolute quantification of cellular levels of photosynthesis-related proteins in Synechocystis sp. PCC 6803. *Photosynth. Res.* 155:219–245.

106. Elbaum-Garfinkle, S., Y. Kim, …, C. P. Brangwynne. 2015. The disordered P granule protein LAF-1 drives phase separation into droplets with tunable viscosity and dynamics. *Proc. Natl. Acad. Sci. USA.* 112:7189–7194.

107. Soding, J., D. Zwicker, …, J. Kirschbaum. 2020. Mechanisms for Active Regulation of Biomolecular Condensates. *Trends Cell Biol.* 30:4–14.

108. Riback, J. A., C. D. Katanski, …, D. A. Drummond. 2017. Stress-Triggered Phase Separation Is an Adaptive, Evolutionarily Tuned Response. *Cell.* 168:1028–1040.e19.

Biophysical Journal, Volume *125*

Supplemental information

The cyanobacterial ESCRT-III protein IM30 forms biomolecular condensates at physiologically relevant conditions

Ndjali Quarta, Tika Ram Bhandari, Katrin Debrich, Nadja Hellmann, Martin Girard, and Dirk Schneider

Supporting information

The cyanobacterial ESCRT-III protein IM30 forms biomolecular condensates at physiologically relevant conditions

Ndjali Quarta,[1] Tika Ram Bhandari,[2] Katrin Debrich[1], Nadja Hellmann,[1] Martin Girard,[2] and Dirk Schneider,[1,3,*]

[1]Department of Chemistry – Biochemistry, Johannes Gutenberg University, 55128 Mainz, Germany

[2]Max Planck Institute for Polymer Research, Ackermannweg 10, Mainz 55128, Germany

[3]Institute of Molecular Physiology, Johannes Gutenberg University, 55099 Mainz, Germany

* Correspondence: Dirk.Schneider@uni-mainz.de

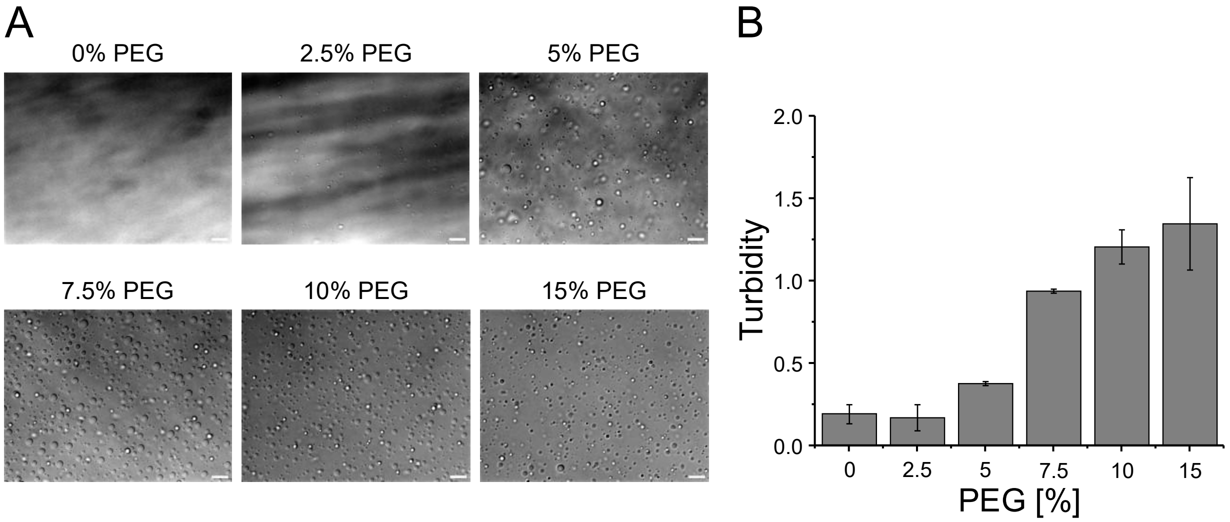

Figure S1: Phase separation and the formation of IM30* condensates at increasing PEG concentrations.

Increasing PEG amounts were added to 32 µM IM30* dissolved in 20 mM HEPES buffer (pH 7.6) containing 100 mM NaCl. The samples were incubated for 5 min. before (A) condensate formation was visualized via DIC microscopy and (B) the turbidity of the solution was measured. At 5% PEG the formation of condensates was already observed, which however, was more pronounced at higher PEG concentrations. Scale bar in (A): 10 µm

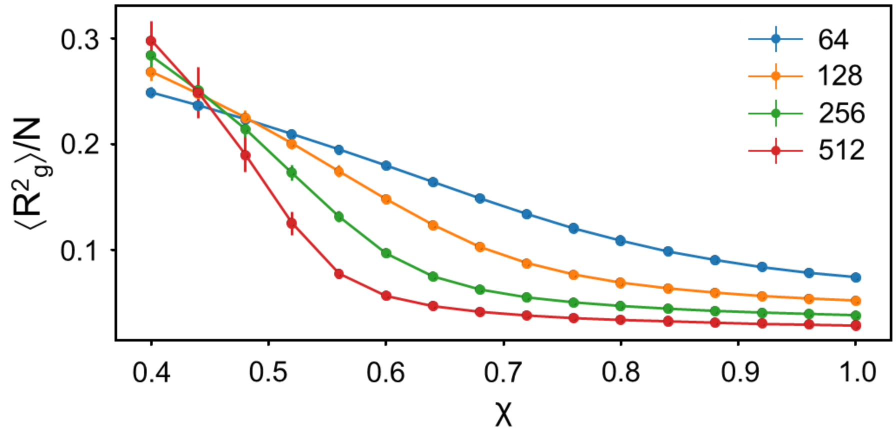

Figure S2: Estimation of the polymer collapse temperature.

The theta point is a special temperature at which a polymer chain in solution behaves ideally, meaning that the chain neither collapses nor expands due to interactions with the surrounding solvent. It also corresponds to the temperature at which an infinite chain goes from an extended conformation to a collapsed one, and, therefore, corresponds to the polymer collapse temperature. At this point, the attractive and repulsive forces between different parts of the chain balance out perfectly.

The theta point can be estimated via computer simulations of chains of different lengths and measuring their radius of gyration, which reflects how spread out the chain is, at different temperatures. When this size measure is normalized and plotted for various chain lengths, the temperature at which the curves cross indicates the theta point. Curves for simulations using different values of a parameter called the Ashbaugh-Hatch scale are shown. The beads are modeled with a size of 6 Å. For these settings, the theta temperature is found to be around 0.45. It’s important to note that this value isn't universal, it rather depends on the chemical details of the polymer, especially the size and nature of its side chains. Changing these features can shift the theta point, so the result is specific to the system being modeled.

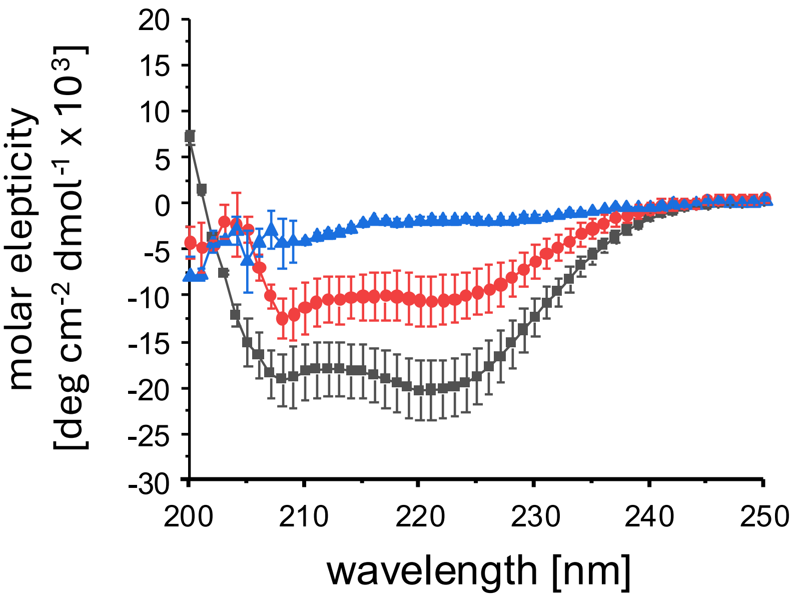

Figure S3: The structure of IM30 wt at increasing urea concentrations.

IM30 wt was incubated in pure buffer (10 mM HEPES pH 7.6; black) or in buffer containing 3 m (red) or 6 M (blue) urea, respectively. After 30 min. incubation, CD spectra were measured. The mean ±SD of three independent measurements (independent protein purifications) is shown. While some α-helix structure is retained at 3 M urea, which corresponds to the helical-hairpin (44), at 6 M urea the protein is completely unfolded.

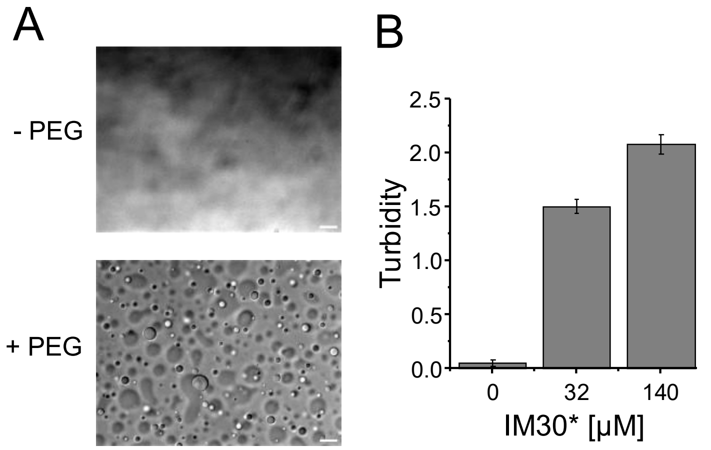

Figure S4: Condensate formation of 140 µM IM30*.

The maximum intracellular IM30 concentration was calculated to be ~140 µM (see main text for details). Even at such high protein concentrations the protein phase separates and form condensates when PEG is present. (A) DIC microscopy images of 140 µM IM30* in 20 mM HEPES buffer (pH 7.6), 100 mM NaCl +/- 10 % PEG. Scale bar: 10 µM. (B) Turbidity of IM30 solutions in the presence of 100 mM NaCl and 10% PEG. Turbidity values for 0, 32 (compare Figure 6 in the main text) and 140 µM protein are shown (n=3 ±SD).
